# Mitochondria-associated endoplasmic reticulum membranes and calcium ion exchange: A novel direction for aging and neurodegenerative diseases

**DOI:** 10.4103/NRR.NRR-D-25-00857

**Published:** 2025-12-30

**Authors:** Yuxuan Yang, Mengjie Chen, Lingling Ding, Jiaxi Liu, Jiansheng Luo, Ruyu Yan, Jiaqi Ning, Siyi Xie, Xiang Li, Zhihao Ren, Ruiling Zhou, Zhuoya Chen

**Affiliations:** 1 Department of Anesthesiology, Beijing Hospital of Traditional Chinese Medicine, Capital Medical University, Beijing, China; 2 Department of Anesthesiology, Deyang People’s Hospital, Deyang, Sichuan Province, China; 3 Department of Acupuncture and Moxibustion, Beijing Pinggu District Hospital of Traditional Chinese Medicine, Beijing, China

**Keywords:** aging, Alzheimer’s disease, amyotrophic lateral sclerosis, calcium channels, frontotemporal dementia, mitochondria-associated endoplasmic reticulum membranes, multiple sclerosis, neurodegenerative diseases, Parkinson’s disease, programmed cell death

## Abstract

Mitochondria-associated endoplasmic reticulum membranes serve as crucial signaling hubs mediating communication between the endoplasmic reticulum and mitochondria, and play a central role in calcium ion exchange. This dynamic interface regulates key cellular processes including bioenergetic metabolism, apoptosis, autophagy, and stress responses. Dysregulation of calcium transport associated with mitochondria-associated endoplasmic reticulum membranes can disrupt intracellular homeostasis, leading to mitochondrial dysfunction, oxidative stress, and neuronal death, which are hallmarks of aging and neurodegenerative diseases. This review systematically examines the functions of protein complexes within mitochondria-associated endoplasmic reticulum membranes and the pathogenic mechanisms of calcium signaling regulated by these membranes in neurodegenerative disorders. It places particular emphasis on structural alterations in calcium ion transport machinery as a common mechanism underlying various neurodegenerative diseases. In Alzheimer’s disease, mitochondria-associated endoplasmic reticulum membranes exhibit a hyperactive state, promoting the generation of amyloid-β and enhancing calcium ion flux from the endoplasmic reticulum to the mitochondria. In contrast, in Parkinson’s disease and amyotrophic lateral sclerosis, the activity of mitochondria-associated endoplasmic reticulum membranes is reduced, leading to a decline in mitochondrial calcium ion buffering capacity and exacerbating excitotoxicity. Proteins residing in mitochondria-associated endoplasmic reticulum membranes are disrupted across various neurodegenerative diseases, resulting in abnormal communication between the endoplasmic reticulum and mitochondria. Recent studies indicate that mitochondria-associated endoplasmic reticulum membranes play a bidirectional role in disease progression, and compensatory mechanisms often exacerbate the pathological process. Therapeutic strategies aimed at preserving the integrity of mitochondria-associated endoplasmic reticulum membranes hold promise for alleviating neurodegenerative damage. Therefore, calcium ion exchange mediated by mitochondria-associated endoplasmic reticulum membranes plays a key role in aging and neurodegenerative diseases, making it a highly promising therapeutic target.

**Facts**

• Mitochondria-associated endoplasmic reticulum membranes are a pathological convergence point where dysregulation of calcium ion homeostasis links various neurodegenerative diseases, including Alzheimer’s disease, Parkinson’s disease, multiple sclerosis, frontotemporal dementia, and amyotrophic lateral sclerosis.

• Structural alterations in core protein complexes associated with mitochondria-associated endoplasmic reticulum membranes can disrupt calcium ion signaling, impair cellular physiological functions, and ultimately drive disease-specific pathological processes.

• Given the importance of mitochondria-associated endoplasmic reticulum membranes structure for cellular physiological functions, therapeutic strategies that directly target this structure face significant challenges. However, treatment approaches aimed at indirectly restoring the integrity and function of mitochondria-associated endoplasmic reticulum membraneshave shown potential for enhancing neuroprotection and promoting neural repair.

**Open questions**

• How do specific protein complexes associated with mitochondria-associated endoplasmic reticulum membranes undergo structural and functional remodeling during different stages of progression in various neurodegenerative diseases?

• How can different subgroups of mitochondria-associated endoplasmic reticulum membranes be selectively regulated in specific brain cell types (such as neurons and glial cells) to achieve selective neuroprotection while maintaining systemic calcium ion homeostasis?

• What are the most reliable targets for restoring the integrity of mitochondria-associated endoplasmic reticulum membranes and calcium ion flux in the pathological processes of neurodegenerative diseases?

## Introduction

Mitochondria-associated endoplasmic reticulum membranes (MAMs) serve as a central signaling hub coordinating intracellular calcium ion (Ca^2+^) homeostasis (Guo et al., 2024). This platform enables direct communication between the endoplasmic reticulum (ER) and mitochondria and provides the foundation for multiple cellular functions, such as Ca^2+^ and reactive oxygen species (ROS) signaling, lipid exchange, mitochondrial dynamics, inflammation, apoptosis, and autophagy (Degechisa et al., 2022).

Ca^2+^ is essential for numerous signaling pathways and cellular functions associated with MAMs. Within MAMs, the primary proteins involved in Ca^2+^ release and recycling are inositol 1,4,5-trisphosphate receptors (IP3Rs) (Li et al., 2024a), which facilitate Ca^2+^ efflux from the endoplasmic reticulum (ER) lumen. Additionally, voltage-dependent anion channel 1 (VDAC1) mediates Ca^2+^ transduction across the outer mitochondrial membrane (Argueti-Ostrovsky et al., 2024). Glucose-regulated protein 75 (GRP75) functions as a chaperone between these two membrane channels (Lemos et al., 2024), with all three proteins localized to the MAMs structure. Furthermore, IP3Rs not only function as Ca^2+^ transport systems within MAMs but also serve as ER-mitochondria tethering proteins, thereby promoting the formation of ER-mitochondrial contact sites (Atakpa-Adaji and Ivanova, 2023). In MAMs, the Sigma-1 receptor (Sig-1R) has garnered significant attention for its regulatory roles in multiple signaling pathways and various mitochondria-associated physiological functions (Munguia-Galaviz et al., 2023). As a highly conserved ER transmembrane protein enriched in MAMs, Sig-1R interacts with proteins involved in Ca^2+^ shuttling and/or ER stress pathway activation (Li et al., 2025c). By stabilizing the conformation of IP3Rs at MAMs, Sig-1R enhances Ca^2+^ efflux from the ER to mitochondria. During ER stress, Sig-1R dissociates from BiP (immunoglobulin-binding protein) and binds to IP3R3, stabilizing these channels at MAMs and promoting Ca^2+^ release from the ER to mitochondria, thereby improving mitochondrial function (Li et al., 2025c). Another key MAMs component is the Parkinson’s disease (PD)-associated DJ-1 protein, which regulates ER-mitochondrial integrity and function by stabilizing the IP3R–GRP75–VDAC protein complex (Basso et al., 2020). Regarding the structural dynamics of MAMs, mitofusins (Mfn1/2) promote contact formation and subsequent mitochondrial membrane fusion through homotypic interactions. Mfn1 localizes exclusively to the outer mitochondrial membrane (OMM), while Mfn2 is present in both the OMM and the ER membrane. The significance of this tethering process and mitochondrial fusion has been documented in PD, amyotrophic lateral sclerosis (ALS), and cancer. In ALS, an alternative ER-mitochondria tethering mechanism involves interactions between vesicle-associated protein B (VAPB) in the ER and protein tyrosine phosphatase-interacting protein 51 (PTPIP51) in the outer mitochondrial membrane (Markovinovic et al., 2024).

Conversely, mitochondrial fission is tightly regulated by the ER, which forms pre-constriction rings on the mitochondrial surface. Fission itself requires the GTPase dynamin-related protein 1 (Drp1), which promotes constriction by forming oligomers and using GTP hydrolysis. However, Drp1 cannot bind the OMM alone to initiate oligomerization; ATPase family AAA domain containing 3A (ATAD3A) facilitates Drp1 binding and thus influences mitochondrial morphology (Zhao et al., 2019b). Additionally, the PD-associated serine/threonine kinase PTEN induced kinase 1 (PINK1) and ubiquitin ligase parkinson protein 2 (Parkin), E3 ubiquitin protein ligase, coordinate ubiquitination of multiple MAM proteins, including VDACand Mfn2, as part of the mitophagy tagging process.

Notably, current research reveals sophisticated regulatory complexity in MAMs-associated signaling, as evidenced by MAMs-mediated calcium pathways in Alzheimer’s disease (AD) study (Wang and Jia, 2023a). Mfn2 not only tethers ER and mitochondria but also regulates Drp1-mediated fission, balancing organelle dynamics. Paradoxically, study on MAM-related proteins such as Mfn2 and Parkin in PD have reported contradictory findings (Parrado-Fernández et al., 2018), highlighting the need for further mechanistic clarification.

Consequently, structural and functional aberrations in MAMs frequently lead to dysregulation of intracellular Ca^2+^ equilibrium, which subsequently triggers aberrant physiological processes and ultimately contributes to disease pathogenesis. Furthermore, various pathological conditions can disrupt intracellular Ca^2+^ homeostasis by inducing structural remodeling of MAMs. This pathological interplay exacerbates disease-related damage, particularly in cardiovascular disorders (e.g., heart failure and atherosclerosis), neurodegenerative diseases (Seegren et al., 2023) (such as AD), and metabolic dysregulations (including insulin resistance), establishing a self-perpetuating cycle of cellular dysfunction.

Substantial gaps persist in the therapeutic targeting of MAMs. Direct inhibitors of critical MAM-resident proteins, such as IP3R, mitochondrial calcium uniporter (MCU), and VDAC1, often carry significant adverse effect risks due to their indispensable roles in fundamental cellular physiological processes (Garbincius and Elrod, 2022). Conversely, targeting MAMs-associated chaperone proteins, exemplified by the Sig-1R, represents a promising therapeutic strategy. This potential is evidenced by the positive phase 3 trial results of the Sig-1R agonist Blarcamesine (ANAVEX2-73), which is now progressing towards regulatory approval.

This review synthesizes groundbreaking insights into MAMs-coordinated Ca^2+^ signaling in neurodegenerative pathologies. We dissect how MAMs dysfunction converges across diseases and propose innovative strategies for targeting MAMs interfaces as a unified therapeutic approach.

## Search Strategy

**Figure 1:**
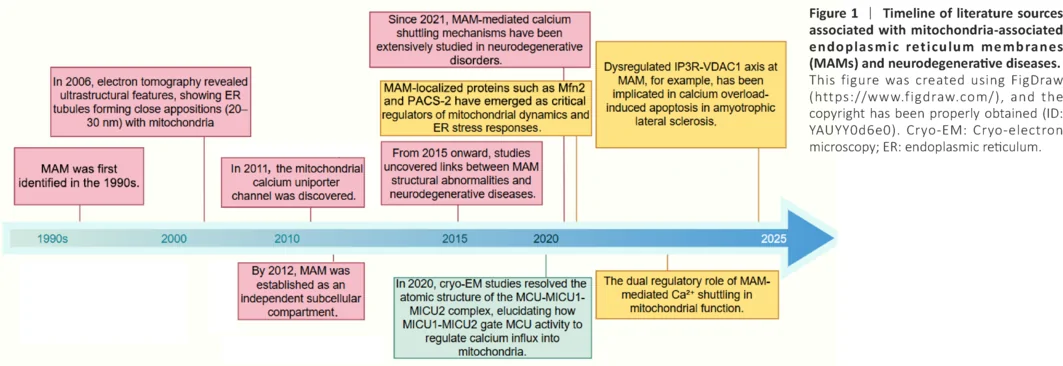
Timeline of literature sources associated with mitochondria-associated endoplasmic reticulum membranes (MAMs) and neurodegenerative diseases. This figure was created using FigDraw (https://www.figdraw.com/), and the copyright has been properly obtained (ID: YAUYY0d6e0). Cryo-EM: Cryo-electron microscopy; ER: endoplasmic reticulum.

A literature search was conducted in the PubMed database for articles published from 1980 to 2025. The search utilized a comprehensive set of keywords including ER-mitochondria crosstalk, apoptosis, autophagy, Alzheimer’s disease (AD), Parkinson’s disease (PD), amyotrophic lateral sclerosis (ALS), multiple sclerosis (MS), frontotemporal dementia (FTD), signal transduction, ER stress, neurodegenerative diseases, mitochondria, Sigma-1 receptor (Sig-1R), inositol 1,4,5-trisphosphate receptor (IP3R), Voltage-dependent anion channel (VDAC), and mitochondrial calcium uniporter (MCU). Relevant studies were identified through various combinations of these terms. Following initial screening of titles and abstracts, full-text articles were assessed for eligibility based on their relevance to the review’s objectives. Ultimately, 199 articles were included in the final analysis, with approximately 90% of the references published within the recent 8-year period (2017–2025). Key milestones and the timeline of major advancements in MAMs-mediated calcium exchange research in neurodegenerative diseases are presented in **Figure 1**.

## Structure and Functions of Mitochondria-associated Endoplasmic Reticulum Membranes

### Structure of mitochondria-associated endoplasmic reticulum membranes

**Figure 2:**
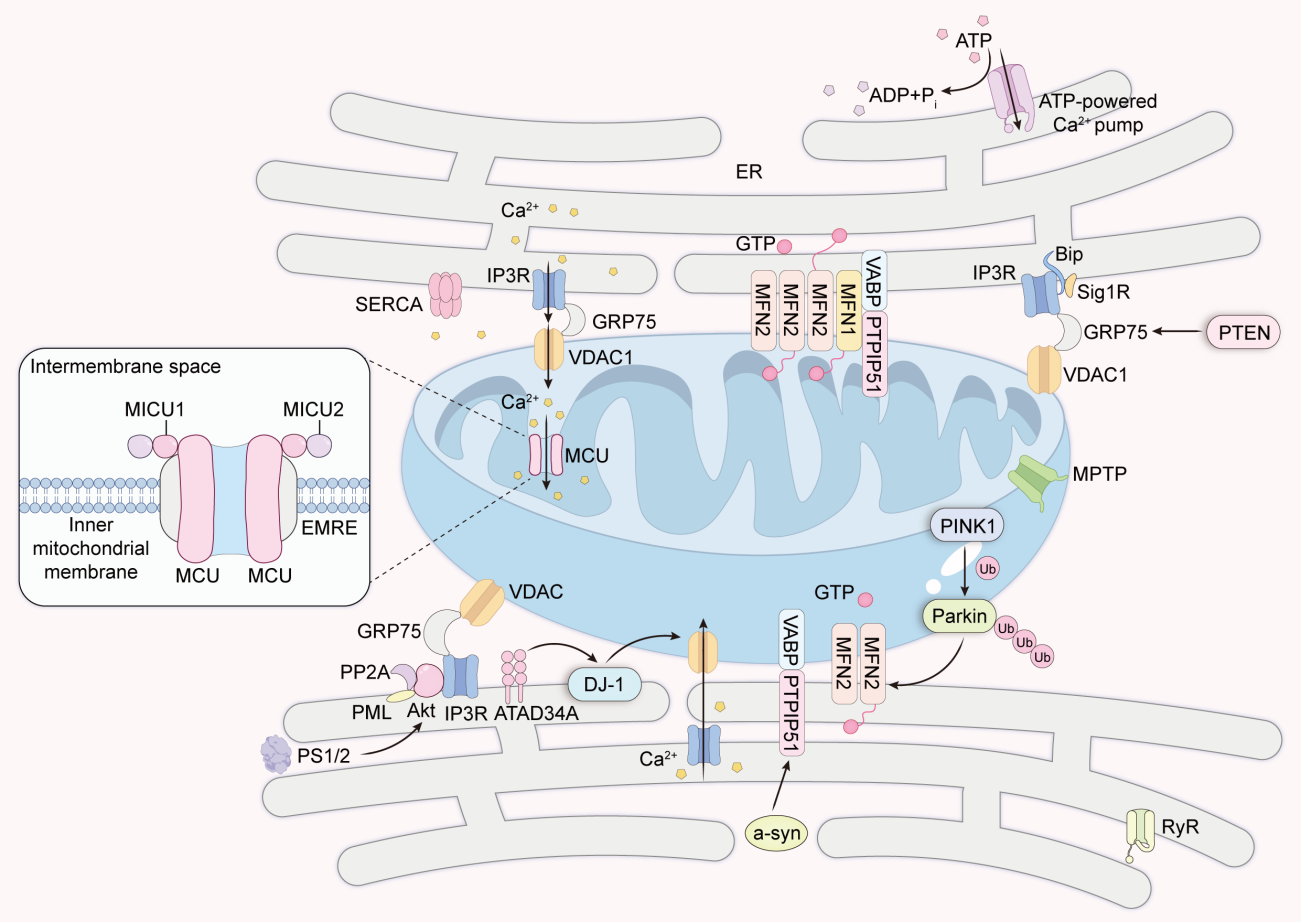
Proteins associated with MAM-mediated calcium exchange. a-Syn: Alpha-synuclein; Akt: protein kinase B; ATAD3A: ATPase family AAA domain containing 3A; Bap31: B-cell receptor-associated protein 31; Bcl-2: B-cell lymphoma-2; Bip: heavy chain binding protein; DJ-1: Parkinson disease protein 7/DJ-1; Fis1: fission 1; FUS: fused in sarcoma; GRP75: glucose-regulated protein 75; GSK3β: glycogen synthase kinase 3β; IRE1α: inositol-requiring enzyme 1α; IP3R: inositol 1,4,5-trisphosphate receptor; MAM: mitochondria-associated endoplasmic reticulum membrane; MCU: mitochondrial calcium uniporter; MFN1/2: mitofusins 1/2; mPTP: mitochondrial membrane permeability transition pore; mTORC1: mammalian target of rapamycin complex 1; PACS2: phosphofurin acidic cluster sorting protein 2; PINK1: PTEN-induced putative kinase 1; PP2A: protein phosphatase 2A; PML: promyelocytic leukemia protein; PS1/2: presenilin 1/2; PTPIP51: protein tyrosine phosphatase-interacting protein 51; ROS: reactive oxygen species; RyR: ryanodine receptor; SERCA: sarcoplasmic/endoplasmic reticulum Ca²⁺ ATPase; Sig-1R: sigma-1 receptor; SOD1: superoxide dismutase 1; TDP-43: TAR DNA binding protein 43; VAPB: vesicle-associated membrane protein associated protein B; VDAC1: voltage-dependent anion channel 1.

MAMs contact the mitochondria and the ER, whose structure is not constant. The spacing width between the outer mitochondria membrane and the ER is about tens of nanometers (the distance between the ER and mitochondria is approximately 10–30 nm according to electron microscopy analysis), as demonstrated by super-resolution microscopy showing 20 nm is the critical distance for Ca^2+^ transfer and mitochondrial oxidative phosphorylation (Dematteis et al., 2024). Proteomic analysis of the structure of MAMs revealed that MAMs contain thousands of proteins responsible for different functions (Zhang et al., 2025b). These key proteins are involved in multiple biological processes, including mitochondrial biogenesis, mitophagy, apoptosis, and various intracellular signal transduction pathways (Zhang et al., 2025a). Furthermore, since the initial discovery of this structure, research on the hub role of MAMs has deepened continuously. MAMs regulate ER and mitochondrial functions, involving numerous physiological processes such as apoptosis and stress, and are widely implicated in neurodegenerative disorders (He et al., 2023). We further discuss the composition of the proteins in MAMs based on their biological roles (**Figure 2** and **Table 1**).

**Table 1:** Proteins localized to the MAMs and their functions

Protein	Location	MAM-related function	Reference
IP3R	ER	Ca^2+^ channel (ER to cytoplasm)	Rizzuto et al., 1998
GRP75	MAM	Regulates Ca^2+^ transport; form MAMs	Rizzuto et al., 1998
VDAC	Mitochondria	Ca^2+^ channel (mitochondria to OMM)	Rizzuto et al., 1998
MFN2	Mitochondria	Regulates mitochondrial dynamics and form MAMs	Tur et al., 2020
PACS2	ER	Regulates Ca^2+^ transport; lipid synthesis	Yu et al., 2019
PINK1	Mitochondria	Involves mitophagy	Quinn et al., 2020
PML	Mitochondria	Recruits PP2A to the IP3R3-Aktcomplex	Pedriali et al., 2017
Parkin	Mitochondria	Involves mitophagy and promotes the ubiquitination of MFN2	Narendra and Youle, 2024
VAPB	Mitochondria	Forms MAMs	Gomez-Suaga et al., 2017
RyR	ER	Ca^2+^ channel (ER to cytoplasm)	Gao et al., 2020
MCU	Mitochondria	Ca^2+^ channel (OMM to IMM)	Liu et al., 2020
PTPIP51	ER	Forms MAMs	Gomez-Suaga et al., 2017
Drp1	Mitochondria	Regulates mitochondrial dynamics	Wang et al., 2025a
PERK	ER	Facilitates the transfer of ROS and promotes the connection between the ER and mitochondria	Smedley et al., 2021
IRE1α	ER	Increases the abundance and stability of IP3R and mediates ER mitochondria dynamics	Shi et al., 2021
Sig-1R	ER	Regulates Ca^2+^ transport and mediates MAM integrity	Shi et al., 2021
α-Syn	Mitochondria	Regulates Ca^2+^ transport and mitochondrial morphology	Vallese et al., 2020
DJ-1	Mitochondria	Promotes the formation of MAMs and facilitates Ca^2+^ transport	Vallese et al., 2020
PS	ER	Forms MAMs, enhances opening of RyR, and stimulates the activity of SERCA	Vallese et al., 2020
APP	ER	Precursor of C99	Vallese et al., 2020
ATAD3A	Mitochondria	Maintains mitochondrial DNA stability and supports mitochondrial protein synthesis	Zhao et al., 2022
C99	ER	Promotes cholesterol transport and increases ceramide production	Area-Gomez and Schon, 2024

APP: Amyloid-beta precursor protein; ATAD3A: ATPase family AAA domain containing 3A; C99: C-terminal fragment of APP; DJ-1: parkinsonism associated deglycase; Drp1: dynamin-related protein 1; ER: endoplasmic reticulum; GRP75: glucose regulated protein 75; IRE1α: inositol-requiring enzyme 1 alpha; MCU: mitochondrial calcium uniporter; MFN2: mitofusin 2; MAM: mitochondria-associated membranes; PACS2: phosphofurin acidic cluster sorting protein 2; PINK1: PTEN induced kinase 1; Parkin: PARK2, E3 ubiquitin protein ligase; PERK: protein kinase RNA-like endoplasmic reticulum kinase; PML: promyelocytic leukemia protein; PTPIP51: protein tyrosine phosphatase interacting protein 51; RyR: ryanodine receptor; SERCA: sarcoplasmic/endoplasmic reticulum calcium ATPase; Sig-1R: sigma-1 receptor; VAPB: vesicle-associated membrane protein-associated protein B; VDAC: voltage-dependent anion channel; α-Syn: alpha-synuclein.

### Calcium ion transport

#### Calcium ion transport and related proteins

The heterogeneous protein components localized on MAMs are critical for maintaining MAMs architecture and functional integrity. Moreover, these proteins actively engage inmultifaceted cellular processes, including intracellular Ca^2+^ trafficking, cholesterol biosynthesis and transport, autophagy, apoptosis, and mitochondrial dynamics regulation. Among these functions, MAMs-associated Ca^2+^ flux regulation is paramount for maintaining normal cellular physiology and exhibits intricate interconnections with other MAMs-orchestrated processes. Dysregulation of this Ca^2+^-handling machinery initiates a pathological cascade linked to diverse disorders, including cardiovascular pathologies, malignant neoplasms, and neurodegenerative disorders.

*Proteins on the endoplasmic reticulum side*

The ER harbors multiple resident proteins critically involved in Ca^2+^ mobilization, particularly in relation to MAMs. Notably, IP3R and the sarco/endoplasmic reticulum Ca^2+^-ATPase (SERCA) emerge as principal mediators within this molecular cohort, orchestrating bidirectional Ca^2+^ flux across ER membranes.

SERCA, a polytopic transmembrane protein, comprises 10 transmembrane α-helices forming its membrane-spanning core. As a prototypical member of the P-type ATPase superfamily, a class of cation-transporting ATPases that utilize adenosine triphosphate (ATP) hydrolysis to drive active ion translocation across biological membranes—SERCA specifically mediates ATP-dependent Ca^2+^ sequestration from the cytosol into the ER/sarcoplasmic reticulum lumen via conformational cycling of its cytoplasmic actuator domain (Li et al., 2025a). To date, 12 SERCA isoforms (SERCA1a/1b, SERCA2a-2d, and SERCA3a-3f) have been molecularly characterized in vertebrates, exhibiting divergent spatiotemporal expression profiles across tissues and developmental stages (Ge et al., 2024). SERCA2b, the constitutively expressed isoform with evolutionarily conserved Ca^2+^-binding affinity, serves as the principal regulator of ER Ca^2+^ refilling and is indispensable for MAM-mediated Ca^2+^ homeostasis.

IP3R, a non-selective cation channel with intrinsic conductivity, serves as a critical node for Ca^2+^ efflux from the ER. This tetrameric channel is assembled from four homologous subunits, with three functionally distinct isoforms—IP3R1, IP3R2, and IP3R3—identified in mammals (Smith et al., 2023). These isoforms exhibit divergent spatiotemporal expression profiles: IP3R1 predominates in neuronal populations, IP3R2 is enriched in myocytes and hepatocytes, while IP3R3 demonstrates broad tissue distribution across diverse cell types. Notably, IP3R localizes to MAMs domains, where it forms a functional tripartite complex with GRP75 and VDAC (Cartes-Saavedra et al., 2025). This supercomplex is essential for MAM-mediated calcium ion transfer. It functions not only as a channel facilitating Ca^2+^ transport from the endoplasmic reticulum to mitochondria but also stabilizes the architecture of MAMs. Its structural integrity directly influences MAM functionality and global calcium homeostasis (Zhang et al., 2025b).

*Proteins on the mitochondrial side*

Upon release from the ER, Ca^2+^ is channeled into the mitochondrial intermembrane space through the VDAC localized on the OMM, followed by spatiotemporally regulated transport into the mitochondrial matrix via the MCU.

VDAC, a conserved β-barrel pore-forming protein, is constitutively expressed in the OMM of eukaryotic mitochondria. (Yang et al., 2024). VDAC selectively permits the passage of water-soluble metabolites and ions while restricting mitochondrial protein translocation. Additionally, it facilitates the transport of diverse molecules, including respiratory substrates, ATP, ROS, mitochondrial DNA, and cytochrome c (Cyt c) (Kmita et al., 2023). Typically, VDAC predominantly operates in two distinct gating states: open and closed conformations. These conformational transitions are dynamically regulated by multiple physiological modulators, including mitochondrial membrane potential (ΔΨm) (Hoogerheide et al., 2022), B-cell lymphoma-2 (Bcl-2) protein family members (Morris et al., 2021), and ambient Ca^2+^ concentrations. Among these modulators, the transmembrane electrochemical potential gradient exerts predominant regulatory control over VDAC channel conductance. Three VDAC isoforms (VDAC1, VDAC2, and VDAC3) have been molecularly characterized in mammals. Notably, VDAC1 has emerged as the most extensively studied isoform, demonstrating critical regulatory roles in mediating OMMCa^2+^ fluxes and orchestrating MAMs -associated Ca^2+^ flux. The VDAC makes a distinct contribution to the pathogenesis of AD. A potential mechanism involves its interaction with disease-specific pathogenic proteins, which triggers mitochondrial dysfunction (Hibino et al., 2023). Meanwhile, specific post-translational modifications of VDAC in ALS model cells were identified via high-resolution mass spectrometry in the context of ALS (Pittalà et al., 2022).

Mitofusin 2 (MFN2), a GTPase protein located on the mitochondrial outer membrane, is crucial for regulating both mitochondrial fusion and the transfer of calcium ions from the ER to the mitochondria (Lv et al., 2025). Mechanistically, Mfn2 tethers mitochondria to the ER by remodeling ER architecture and functioning as a membrane tether (Naón et al., 2023). However, its precise role in MAMbiogenesis and facilitation of ER-mitochondria contact sites remains subject to divergent mechanistic interpretations (Cefis et al., 2024). Contrary to previous assumptions, Cieri et al. (2018) discovered that acute downregulation of Mitofusin 2 in HeLa cells resulted in an increase in short-range mitochondria-ER contacts. Paradoxically, under the same conditions of MFN2 downregulation, the number of long-range ER-mitochondria interactions was also significantly elevated. This seemingly contradictory phenomenon suggests that MFN2 modulates the frequency of ER-mitochondria contacts without altering their spatial distribution or temporal dynamics. Consequently, MFN2 is dispensable for the formation and maintenance of ER-mitochondria junctions and MAMs, challenging its established role as a critical tethering component. Experimental evidence confirms MFN2 neither constitutes a core structural element of MAM tethering complexes nor directly enables MAM assembly. Instead, it serves as a negative regulator that constrains excessive organelle contact under specific conditions to prevent cellular damage (Filadi et al., 2015). This regulatory role is crucial for cell survival, as it restricts stimulus-induced hyperconnectivity between the ER and mitochondria, thereby reducing detrimental Ca^2+^ transfer to mitochondria and safeguarding cells against potential damage.

The mitochondrial permeability transition pore (mPTP), a large non-selective channel within the inner mitochondrial membrane (IMM) (Bernardi et al., 2023), demonstrates preferential localization or enrichment at mitochondrial contact sites where the IMM and outer mitochondrial membrane (OMM) form tight junctions. This strategic positioning enables pore opening to directly compromise OMM integrity. Although the precise 3D architecture of mPTP remains unresolved, it is conceptualized as a dynamic multiprotein complex with variable pore diameter. Its molecular composition remains incompletely defined. Functionally, mPTP operates as a permeability switch for the IMM: In the closed state, it maintains IMM impermeability to protons, ions, and metabolites—essential for preserving mitochondrial membrane potential (ΔΨm) and ATP synthesis. Upon opening, the IMM becomes transiently permeable to solutes ≤ 1.5 kDa (including protons, ions, metabolites, and select proteins) (Zhu et al., 2024b). Sustained mPTP opening serves as the core mechanism in multiple cell death pathways (Bernardi et al., 2023). Although not structurally embedded within MAMs, this entity functionally integrates with MAMs-regulated calcium signaling pathways. Critically, emerging evidence implicates dysregulated mPTP activity in neurodegenerative pathogenesis (Vanderhaeghe et al., 2024).

**Figure 3:**
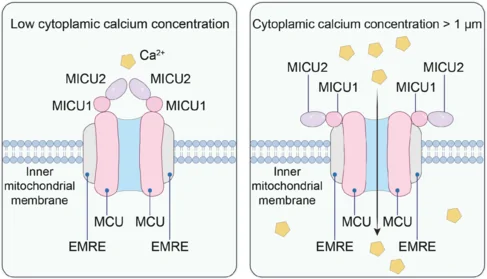
Structural and functional properties of the MCU protein. Within the mitochondrial intermembrane space, MICU1 and MICU2 form disulfide-bonded dimers. MICU1 enhances the open probability of the MCU, while MICU2 imposes a steric blockade on the MCU pore. Under resting Ca²⁺ conditions, MICU1-MICU2 heterodimers promote MCU channel closure through MICU2-mediated suppression. Upon elevated Ca²⁺ concentrations, conformational rearrangement of the MICU1-MICU2 complex relieves MICU2-dependent inhibition and potentiates MICU1-driven MCU activation. EMRE: Essential MCU regulator; MCU: mitochondrial calcium uniporter; MICU1: Mitochondrial calcium uptake protein 1; MICU2: mitochondrial calcium uptake protein 2.

The IMM, distinct from the ion-permeable OMM, imposes stringent selectivity against charged ion diffusion. This electrochemical barrier necessitates Ca^2+^ entry into the mitochondrial matrix via the MCU—a transmembrane channel complex with low Ca^2+^-binding affinity embedded within the IMM (Li et al., 2023). The MCU macromolecular complex in humans comprises four functionally integrated components: the pore-forming MCU subunit, its dominant-negative isoform MCUb, the essential MCU regulator (EMRE), and the intermembrane space-localized MICU1–MICU2 heterodimeric complex (D’Angelo and Rizzuto, 2023). Structural investigations confirm that mitochondrial calcium uptake 1 (MICU1) and mitochondrial calcium uptake 2 (MICU2) form covalent homodimers throughdisulfide bonds. The MCU channel exhibits a unique pentameric “vase-shaped” architecture, as resolved by using cryo-electron microscopy (cryo-EM) and nuclear magnetic resonancespectroscopy. Recent cryo-EM studies reveal that the functional homocomplex assembles in an 8:8:2:2 stoichiometry (MCU:EMRE:MICU1:MICU2), where EMRE anchors MICU1–MICU2 to the MCU pore, forming a “cap-like” regulatory module (De Mario et al., 2023). Functional characterization using synthetic lipid bilayer models reveals their antagonistic regulatory roles: MICU1 stabilizes the MCU open conformation, whereas MICU2 exerts inhibitory control (Hasan et al., 2024). The MICU1–MICU2 heterodimer functions as a Ca^2+^-sensitive gatekeeper through a dynamic conformational switch. Under physiological resting conditions ([Ca^2+^] < 300 nM), MICU2 dominates, sterically occluding the MCU pore via disulfide-bond-stabilized dimerization, preventing untimely Ca^2+^ influx and mitochondrial overload. Elevated cytosolic Ca^2+^ levels (> 1 μM) trigger Ca^2+^-dependent conformational switching of the MICU1–MICU2 complex (Rodríguez-Prados et al., 2023). Ca^2+^ binding to EF-hand domains triggers a structural rearrangement. MICU1 undergoes an activation transition (Kaye et al., 2024), displacing MICU2 and promoting pore opening, while MICU2’s inhibition isalleviated (Yoo, 2022). This cooperative mechanism enhances channel open probability by > 10-fold, enabling rapid response to physiological stimuli like ER-mitochondrial Ca^2+^ microdomains, creating a bimodal regulatory mechanism for mitochondrial Ca^2+^ homeostasis (**Figure 3**).

## Mitochondria-associated Endoplasmic Reticulum Membrane-associated Calcium Transport: Integrative Roles in Cellular Functions and Interorganellar Crosstalk

**Figure 4:**
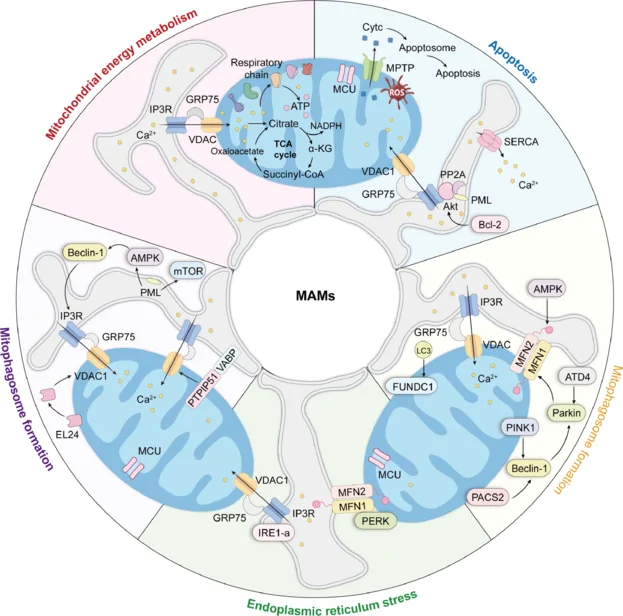
Effects of MAM-related calcium transport on cellular function. Mitochondrial energy metabolism: MAMs assist mitochondria in acquiring Ca^2+^. The MCU elevates matrix Ca^2+^, and energy transduction is mediated by enzymes such as PDH, ICDH, and OGDH. Ca^2+^ also activates ATP synthase to promote ATP production. Endoplasmic reticulum stress: IRE1α and PERK, located in MAMs, mediate the unfolded protein response, regulate the structure of MAMs, and enhance energy output. They regulate inter-organelle communication, control IP3R distribution, stabilize complexes, and participate in cell survival regulation. Ubiquitination by MITOL inhibits its pro-apoptotic effect. It acts as a molecular intermediary during reactive oxygen species-induced stress, initiating apoptosis via the signaling axis, promoting organelle coupling and ROS transmission, regulating mitochondrial dynamics and morphological plasticity, and interacting with LRRK2 to affect MAM abundance. Apoptosis: Ca^2+^ transport regulated by MAMs modulates apoptosis signaling. Excess Ca^2+^ activates mPTP, causing a collapse of mitochondrial membrane potential and impairment of ATP synthesis. The rupture of the OMM releases pro-apoptotic factors. Autophagy and mitophagy: Dysregulated Ca^2+^ levels in MAMs trigger abnormal autophagy. EI24 interacts with the transport complex to maintain MAM structure and facilitate Ca^2+^ shuttling. Interrupted Ca^2+^ flux recruits AMPK to initiate autophagy. The VAPB-PTPIP51 complex optimizes Ca^2+^ uptake; its deficiency leads to autophagic abnormalities. MAMs coordinate mitophagy via the PINK1/Parkin, FUNDC1, and PACS2 axes. AMPK: AMP-activated protein kinase; ICDH: isocitrate dehydrogenase; IRE1α: inositol-requiring enzyme 1 alpha; MAM: mitochondria-associated endoplasmic reticulum membrane; MCU: mitochondrial calcium uniporter; MITOL: mitochondrial E3 ubiquitin ligase; OGDH: oxoglutarate dehydrogenase; OMM: outer mitochondrial membrane; PDH: pyruvate dehydrogenase; mPTP: mitochondrial permeability transition pore; PINK1: PTEN-induced kinase 1; PACS2: phosphofurin acidic-cluster sorting protein 2; PERK: protein kinase RNA-like endoplasmic reticulum kinase; ROS: reactive oxygen species; VAPB: vesicle-associated membrane protein B; PTPIP51: protein tyrosine phosphatase interacting protein 51; IP3R: inositol trisphosphate receptor.

Interorganellar crosstalk refers to the dynamic signal transmission and functional coordination between subcellular structures, enabling cells to integrate physiological processes such as metabolism, stress responses, and fate determination. MAMs form specialized contact sites that function as critical platforms for these cellular processes; these structures are termed membrane contact sites (MCS). Central to this coordination is calcium exchange mediated by IP₃R-VDAC interactions facilitated by molecular chaperones such as GRP75. In neurodegenerative diseases, MAMs are essential for facilitating mitochondrial-ER communication. Dysregulation of mitochondrial-ER interactions is increasingly recognized as a contributing factor in the pathogenesis of multiple neurological disorders (**Figure 4**).

### Mitochondrial energy metabolism

Mitochondria are signaling organelles that regulate multiple cellular functions and determine cell fate. Their tricarboxylic acid cycle intermediates, often considered crucial for biosynthetic purposes, also act as signaling molecules, controlling chromatin modification, DNA methylation, hypoxic responses, and immunity (Todorova et al., 2023). A recent study has identified that mitochondrial metabolic abnormalities caused by alterations in MAM function may underlie the bioenergetic deficits observed in ALS (Larrea et al., 2025). Mitochondria acquire cytosolic Ca^2+^ via MAMs—critical membrane contact sites that coordinate cellular bioenergetics and apoptosis, both indispensable intracellular processes. Substantial evidence demonstrates that the MCU transiently elevates matrix Ca^2+^ to physiological thresholds, thereby amplifying mitochondrial energy transduction. This metabolic regulation is primarily mediated by three key mitochondrial matrix enzymes essential for aerobic oxidation: pyruvate dehydrogenase complex, isocitrate dehydrogenase, and α-ketoglutarate dehydrogenase complex (OGDH). These enzymes are allosterically regulated by intramitochondrial Ca^2+^ concentrations. Additionally, mitochondrial Ca^2+^ directly activates F_0_F_1_-ATP synthase in the electron transport chain, thereby enhancing ATP production through oxidative phosphorylation (OXPHOS) (Popov, 2023). Induced pluripotent stem cells (iPSC)–derived neurons from patients with ALS exhibited increased MAM contact site density but demonstrated functional defects, resulting in impaired Ca^2+^ buffering capacity. This triggered oxidative stress, leading to mitochondrial calcium overload and compromised ATP synthesis (Denley et al., 2025).

### Endoplasmic reticulum stress

The endoplasmic reticulum (ER) is a critical organelle responsible for protein quality control and cellular homeostasis (Yang et al., 2025). Endoplasmic reticulum stress (ERS) serves as both a hallmark and underlying mechanism in numerous neurodegenerative disorders, while Ca^2+^ signaling plays a pivotal role in orchestrating stress responses (Singh et al., 2024). As two pivotal ER stress transducers, inositol-requiring enzyme 1α (IRE1α) and protein kinase R-like endoplasmic reticulum kinase (PERK) exhibit colocalization within MAMs. These activated sensors not only mediate canonical unfolded protein response (UPR) pathways but also functionally regulate MAMs architecture through dynamic interactions with multiple MAM-resident proteins. Such regulatory mechanisms strengthen ER-mitochondria physical coupling, thereby potentiating mitochondrial bioenergetic output through enhanced ATP synthesis and calcium ion sequestration. Intriguingly, emerging evidence reveals non-canonical, stress-independent functions for IRE1α and PERK in MAM-mediated cellular processes (Zhao and Sheng, 2025).

The ERS sensor IRE1α demonstrates particular functional significance in modulating MAM-mediated inter-organelle communication. A mechanistic study delineates a biphasic attenuation pattern of IRE1α enzymatic activity: initial autophosphorylation triggers partial inactivation of its ribonuclease domain, while complete functional termination requires the re-establishment of ER-mitochondria membrane contacts following stress-induced organelle separation (Pelizzari Raymundo et al., 2022). The protein kinase B-mechanistic target of rapamycin (AKT-mTOR) signaling cascade emerges as a critical regulator of contact site reformation during ERS recovery (Siwecka et al., 2021). Furthermore, accumulating evidence establishes stress-unrelated physiological roles of IRE1α within MAM microdomains. Notably, a recent study demonstrates IRE1α-mediated regulation of IP3R spatial distribution across ER subdomains (Zhao and Sheng, 2025). A novel mechanistic model proposes that IRE1α functions as a structural scaffold within MAMs, physically stabilizing IP3R complexes through direct protein interaction. This scaffolding capacity enhances IP3R protein stability and microdomain retention, thereby fine-tuning calcium flux dynamics at ER-mitochondria interfaces. These findings collectively establish a paradigm-shifting role for IRE1α as a molecular platform governing calcium channel organization in membrane contact sites. In fact, IRE1α in MAMs not only governs ER-mitochondria dynamics and mitochondrial calcium uptake but also plays a pivotal role in regulating cell survival. The mitochondrial ubiquitin ligase MITOL (also known as MARCH5) has been shown to inhibit ER stress-induced apoptosis by ubiquitinating IRE1α within MAMs. Furthermore, MITOL can ubiquitinate another MAM protein, Mitofusin 2, thereby enhancing its GTPase activity and promoting the interaction between the ER and mitochondria (Takeda et al., 2019).

As another critical MAM-resident protein implicated in ER stress responses, PERK serves as a molecular intermediary at ER-mitochondria contact sites during ROS-induced ERS (Bassot et al., 2023). Through the canonical PERK-eIF2α-ATF4 signaling axis, PERK activates C/EBP homologous protein (CHOP), thereby initiating mitochondrial apoptosis via upregulation of BH3-only proteins such as Bim, which subsequently triggers Bax activation (Verfaillie et al., 2012). Concurrently, MAM-localized PERK facilitates physical coupling between ER and mitochondria, enabling rapid reactive oxygen species transmission in the form of lipid hydroperoxides across organelles (Li et al., 2025b). This interorganellar signaling cascade drives massive Ca^2+^ efflux from ER to mitochondria, ultimately inducing mitochondrial phospholipid oxidation and cytochrome c (Cyt c) release from ER-proximal mitochondrial pools. Sustained phospholipid oxidation promotes Bax insertion into the mitochondrial outer membrane, culminating in full apoptotic commitment. Emerging evidence highlights the regulatory roles of PERK in mitochondrial dynamics (Perea et al., 2023). Mitofusin 2, a recognized PERK-interacting partner, demonstrates functional reciprocity within MAMs: Mitofusin 2 binding suppresses PERK activity, whereas Mitofusin 2 deficiency induces mitochondrial dysfunction through persistent PERK activation. An early study has found that Mitofusin 2 can regulate ERS and restore mitochondrial function via MAM (Zhu et al., 2024a). The Mfn2-PERK axis also interfaces with microRNA regulatory networks. During ERS, PERK-dependent transcription factors ATF4 and Nrf2 repress the miR-106b-25 cluster Mitofusin 2. PERK modulates mitochondrial fission dynamics through miR-106b suppression in MAMs under stress conditions. A recent mechanistic study revealed that di(2-ethylhexyl) phthalate (DEHP) induces ERS via activation of the PERK/eIF2α/ATF4 pathway while concurrently impairing MAM integrity through PERK-mediated dysregulation of Mitofusin 2, ultimately contributing to neurotoxicity (Zhao et al., 2024). Furthermore, PERK coordinates mitochondrial morphological plasticity during ERS by regulating stress-induced mitochondrial hyperfusion. Depletion of the ATP-dependent protease YME1L enhances PERK-driven stress-induced mitochondrial hyperfusion, facilitating dynamic mitochondrial network remodeling (Lebeau et al., 2018). Intriguingly, PERK-mediated ubiquitination pathways directly interact with leucine-rich repeat kinase 2 (LRRK2), a MAM constituent that regulates ER-mitochondria tethering and bioenergetics. LRRK2 kinase activity modulates E3 ubiquitin ligases (MARCH5, MULAN, Parkin), with kinase-inactive LRRK2 variants triggering PERK-dependent phosphorylation. This post-translational modification induces ubiquitin-mediated degradation of ER-mitochondria tethering proteins, ultimately reducing MAM abundance (Toyofuku et al., 2020).

### Apoptosis

Apoptosis, or programmed cell death, constitutes a tightly regulated biological process critical for maintaining physiological homeostasis (Newton et al., 2024). Ca^2+^ transport mechanisms governed by MAMs regulatory genes play indispensable roles in modulating apoptotic signaling cascades (Larrañaga-SanMiguel et al., 2025). Experimental evidence demonstrates that exceeding threshold Ca^2+^concentrations within the mitochondrial matrix initiates mPTP activation. Sustained mPTP opening induces mitochondrial membrane permeability transition, characterized by collapsed mitochondrial membrane potential (ΔΨm) and consequent impairment of ATP biosynthesis, which is closely associated with brain aging and AD (Waseem and Wang, 2023). Additionally, this process results in alterations in mitochondrial osmotic pressure, potentially leading to the rupture of the outer mitochondrial membrane. The inhibition of ATP production due to mitochondrial membrane perturbations disrupts cytoplasmic ion homeostasis, which can ultimately lead to cell necrosis. Moreover, the rupture of the outer mitochondrial membrane triggers the release of various pro-apoptotic factors from the mitochondria (Sun et al., 2025). Aberrant opening of the MPTP induces mitochondrial matrix swelling and outer membrane rupture, precipitating the release of pro-apoptotic factors such as Cyt c and triggering apoptosis (Pukoli and Vécsei, 2023). Although this pore is not structurally embedded within MAMs, it is functionally coupled to the calcium signaling pathways regulated by MAMs. Within MAMs microdomains, the IP3R-GRP75-VDAC1 complex-mediated Ca^2+^ influx is recognized as a crucial conduit for calcium overload during apoptotic cascades (Wang et al., 2021). In rodent models of severe traumatic brain injury, neuroinflammatory responses and ERS-mediated apoptosis are significantly correlated with altered expression of proteins governing ER-mitochondrial coupling and calcium transfer (Chen et al., 2022).

### Autophagy

Dysregulated Ca^2+^ signaling within MAMs may instigate aberrant autophagic activity, which is associated with aging and the development and progression of diseases. Etoposide-induced protein 24 (EI24), an ER-resident protein implicated in autophagy regulation, undergoes stress-induced translocation to MAMs microdomains. At these inter-organelle interfaces, EI24 directly engages with the Ca^2+^-transporting core complex comprising IP3R, GRP75, and VDAC. This tripartite interaction is critical for preserving MAMs ultrastructural integrity and modulating Ca^2+^ shuttling efficiency (Xu et al., 2022). Mechanistically, disrupted ER-to-mitochondria Ca^2+^ flux triggers AMP-activated protein kinase recruitment to MAMs (Hu et al., 2021). The translocated AMP-activated protein kinase subsequently initiates autophagosome biogenesis via phosphorylation of Unc-51-like autophagy activating kinase 1 (ULK1) within the Beclin-1 signaling axis (Danese et al., 2017). The vesicle-associated membrane protein-associated protein B (VAPB)-PTPIP51 tethering complex enhances IP3R-VDAC coupling at MAMs (Obara et al., 2024), thereby optimizing mitochondrial Ca^2+^ uptake. Genetic ablation of either VAPB or PTPIP51 destabilizes MAM architecture, resulting in two distinct pathological outcomes: (1) elevated cytosolic Ca^2+^ accumulation accompanied by increased autophagic flux, and (2) mitochondrial depletion due to enhanced organelle autophagy (mitophagy). These findings collectively demonstrate that the VAPB-PTPIP51 axis suppresses excessive autophagy by maintaining IP3R-mediated mitochondrial Ca^2+^ homeostasis.

Notably, MAMs orchestrate intracellular mitophagy through three distinct molecular axes: PINK1/Parkin pathway, FUNDC1 pathway, and PACS2 pathway. For example, mitochondrial Lon protease—a multifunctional, stress-inducible chaperone critical for proteostasis and stress adaptation—physically interacts with the MAM-localized Na^+^/Ca^2+^ exchanger to mediate Ca^2+^ efflux. This Ca^2+^-dependent signaling cascade activates the FUNDC1-ULK1 mitophagy axis, thereby enhancing cellular survival under stress conditions (Ponneri Babuharisankar et al., 2023). Concurrently, PACS2 coordinates mitochondrial quality control through two main functions: (1) preserving MAM structural integrity by maintaining the tethering between the ER and mitochondria, and (2) redistributing Beclin-1 to MAM microdomains (Li et al., 2022). This spatial reorganization enhances mitochondrial Ca^2+^ shuttling efficiency and initiates selective autophagy via the recruitment of the Beclin-1-containing class III phosphatidylinositol 3-kinase complex, which promotes mitophagy through the phosphatidylinositol 3-kinase/AKT/mTOR pathway (Zheng et al., 2021). Experimental evidence further demonstrates that MAMs serve as central regulatory platforms for mitochondrial quality surveillance, with mitophagy modulation emerging as their primary function. Genetic or pharmacological disruption of MAM integrity disrupts mitophagic flux, resulting in either pathological organelle accumulation or excessive mitochondrial clearance.

Key nodal regulators functionally integrate multiple signaling cascades through coordinated molecular cross-talk. Ethanol exposure triggers ERS and apoptosis in neuroglial cells by inducing oxidative stress and disrupting Ca^2+^ homeostasis. This manifests as significant upregulation of pro-apoptotic p53 and executioner caspases, concomitant with downregulation of anti-apoptotic Bcl-2 (Sushma et al., 2023). Concurrently, ligand-activated death receptors initiate transcription of ERS-responsive pro-apoptotic BCL-2 proteins (e.g., BIM, PUMA), facilitating mitochondrial Cyt c release. Both IRE1 and PERK branches of the UPR converge on apoptosis induction during ER stress. Central to this regulation is CHOP—a PERK-downstream transcription factor directly activated by ATF4. CHOP-driven expression of growth arrest and DNA damage-inducible 34 recruits protein phosphatase 1 to dephosphorylate phospho-eukaryotic initiation factor 2α, thereby reversing translational attenuation and enabling synthesis of terminal apoptotic effectors (Mao et al., 2022b).

Autophagy, an essential cellular repair mechanism, is dynamically modulated by stress and pathological conditions. Research demonstrates that ER-phagy (selective autophagic clearance of ER components) mitigates acute kidney injury through resolution of ERS and downstream inhibition of apoptotic pathways (Jin et al., 2024). Accumulating evidence reveals an intricate interdependence among mitochondrial oxidative respiration, ERS, autophagy, and apoptotic signaling. Oxidative stress orchestrates ERS-mediated activation of both apoptotic and autophagic pathways (Huang et al., 2020). Conversely, coordinated inhibition of oxidative stress, ERS, and autophagy confers cytoprotection against PM2.5-induced apoptosis in HaCaT keratinocytes (Zhu et al., 2022).

MAMs serve as fundamental structural platforms for interorganellar communication, wherein calcium (Ca^2+^) functions dually as a transported ion and a dynamic signaling molecule to fine-tune physiological cellular processes. Conversely, dysregulation of this organelle crosstalk is mechanistically linked to neurodegenerative pathologies. Architecture-focused investigations of MAM-mediated signaling networks hold significant potential to inspire novel therapeutic targeting strategies.

## Relevance of Mitochondria-associated Endoplasmic Reticulum Membrane-related Calcium Transport in Aging and Neurodegeneration

### Aging

**Figure 5:**
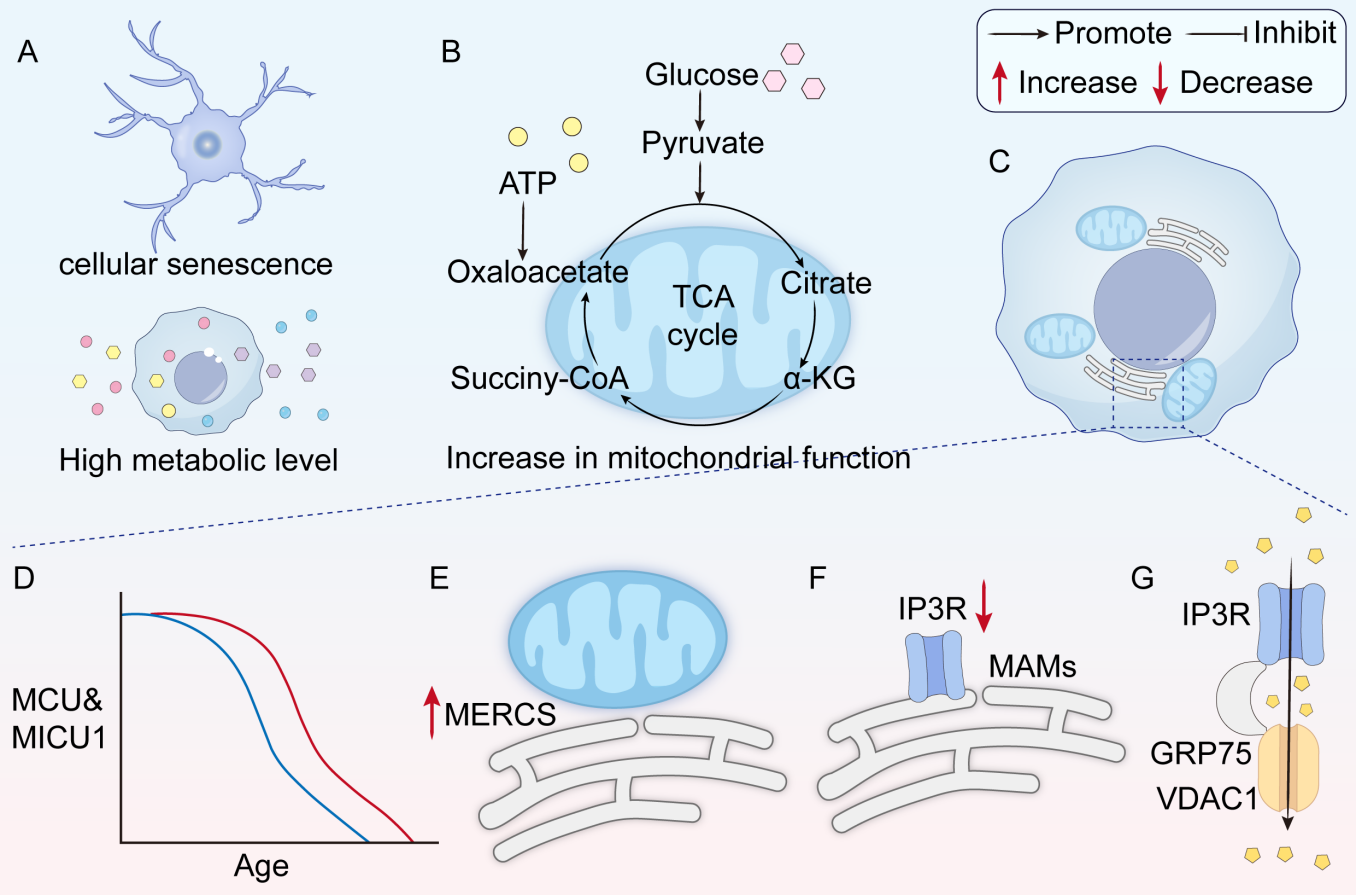
Significant physiological changes in senescent cells. Although physical mitochondria-ER contacts (MERCS) may increase (E) in senescent cells (C), the key molecular complexes mediating calcium ion transfer (e.g., IP3R-VDAC1, G) are reduced in formation, and the expression of the mitochondrial calcium uptake channel (MCU, D) is decreased. These changes ultimately result in diminished calcium ion flux from the endoplasmic reticulum to mitochondria (F). This disruption in calcium ion signaling impairs mitochondrial functions such as energy production (B), and is closely associated with heightened glycolysis (A) and functional abnormalities in senescent cells.

Cellular senescence represents a hallmark of aging, characterized by the progressive accumulation of senescent cells (Melo Dos Santos et al., 2024). Driven by irreversible proliferative arrest, this multifactorial process not only impairs tissue regeneration but also promotes chronic inflammation and metabolic dysregulation, exacerbating age-related pathologies such as neurodegenerative diseases. Neurons, with their exceptionally high bioenergetic demands, are particularly susceptible to mitochondrial dysfunction (**Figure 5**).

A study on senescent cell metabolism has shown increased glucose uptake and lactate production, indicating enhanced glycolysis (Wang et al., 2022). Additionally, senescent cells exhibit higher mitochondrial function (pyruvate oxidation, tricarboxylic acid cycle, and respiration) and mitochondrial mass compared with normally growing cells (Dou et al., 2023). The enhancement in mitochondrial function results from elevated pyruvate dehydrogenase activity, driven by retinoblastoma protein–dependent upregulation of its positive regulator, pyruvate dehydrogenase 2 (Takebayashi et al., 2015). Notably, pyruvate dehydrogenase 2 is Ca^2+^-regulated, and its activity decreases when low-level constitutive inositol trisphosphate receptor (IP3R)-mediated Ca^2+^ transfer to mitochondria is inhibited (Puebla-Huerta et al., 2025). A recent study employing therapy-induced senescence models reveal a paradoxical remodeling of mitochondria-endoplasmic reticulum contact sites: while the contact area expands, ER-to-mitochondria Ca^2+^ transfer is markedly diminished. This discordance arises from therapy-induced senescence-associated downregulation of IP3R expression and disrupted IP3R-VDAC1 complex assembly, effectively decoupling interorganellar Ca^2+^ signaling (Seegren et al., 2023). Furthermore, the age-related decline in mitochondrial calcium uptake protein and its regulatory subunit, MICU1, correlates with diminished mitochondrial Ca^2+^ uptake capacity in senescent cells. This progressive dysregulation of cellular Ca^2+^ homeostasis potentiates chronic low-grade inflammation through unresolved ERS and impaired efferocytosis (Tambini et al., 2016). Mitochondria dynamically regulate Ca^2+^ homeostasis as integrated sensors, buffers, and modulators (Ahumada-Castro et al., 2021). While transient cytosolic Ca^2+^ spikes trigger rapid mitochondrial sequestration to prevent cytosolic overload, sustained elevation induces mitochondrial Ca^2+^ overload. This pathological state drives increased ROS production, impaired ATP synthesis, and activates degenerative cascades, including: Ca^2+^-stimulated nitric oxide generation; cytochrome c dissociation from the inner mitochondrial membrane (IMM); mPTP opening with consequent release of cytochrome c and antioxidant enzymes; arachidonic acid pathway activation; and calcium/calmodulin-dependent kinase II (CaMKII) signaling (Ahumada-Castro et al., 2021). Pathological mitochondrial Ca^2+^ accumulation during aging stems from IP3R-driven ER Ca^2+^ release and MCU-mediated influx, ultimately precipitating collapse of the mitochondrial membrane potential (ΔΨm), elevated ROS generation, and accelerated cellular senescence (Müller et al., 2018).

Moreover, aging-related chronic neuroinflammation represents a key factor contributing to age-associated cognitive impairment, which has been well-studied in the context of neurodegenerative diseases (Heneka et al., 2025). Seegren et al. (2023) revealed that mitochondrial Ca^2+^ uptake and metabolic capacity undergo age-dependent decline in macrophages. This progressive mitochondrial dysfunction disrupts Ca^2+^ signaling homeostasis, thereby driving chronic inflammation implicated in age-associated pathologies.

Brain integrity and function critically depend on mitochondrial oxidative phosphorylation. Although constituting merely 2% of body mass, the brain consumes approximately 20% of the total oxygen supply (Faitg et al., 2021). This exceptional bioenergetic demand subjects cerebral mitochondria to substantial metabolic stress. Given the pivotal role of mitochondria in aging, mitophagy—the selective autophagic clearance of damaged mitochondria coupled with the coordinated biogenesis of functional replacements—constitutes an essential anti-aging mechanism that preserves mitochondrial fitness and activity. By eliminating dysfunctional organelles and enabling neuronal metabolic recovery, the restoration of mitophagic flux facilitates neural repair and neuroregeneration (Lou et al., 2020).

Currently, ERS and mitochondrial dysfunction are increasingly proposed as key therapeutic targets against neuropathology, with MAMs emerging as a crucial intersection for this collaboration. In neurodegenerative diseases, several functions regulated by ER-mitochondrial signaling are disrupted, including bioenergetics and mitochondrial ATP production, Ca^2+^ homeostasis, axonal transport, lipid metabolism, inflammation, autophagy, and the ERS response.

### Interaction between aging and neurodegenerative diseases

**Figure 6:**
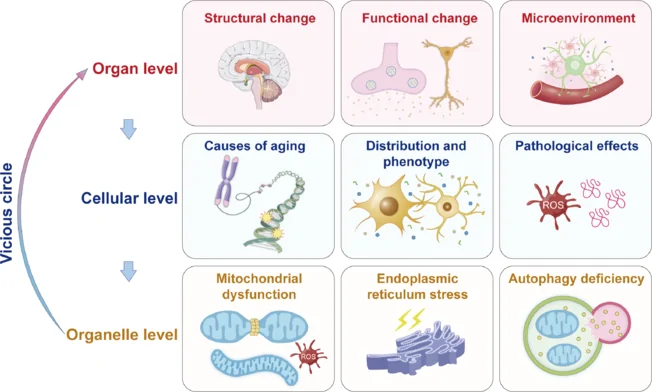
The relationship between aging and neurodegeneration spans hierarchical levels, from organelle to organismal scales. Structural and functional alterations at the organ level (e.g., brain structure, neuronal function, microenvironment), cellular-level factors (causes of aging, phenotype distribution, pathological effects such as reactive oxygen species), and organelle-level dysfunctions (mitochondrial, endoplasmic reticulum, and autophagy deficiencies) interact in a vicious circle, driving nervous system aging.

A profound and complex interplay exists between aging and neurodegenerative disorders, including AD, PD, and ALS. Aging is the primary risk factor for neurodegeneration. Understanding this relationship requires a multiscale analysis that encompasses systemic brain aging, cellular senescence (particularly neuronal senescence), and organellar decline. These processes interact synergistically, establishing the foundation for neurodegenerative pathogenesis (**Figure 6**).

At the organ level, accumulated damage during brain aging—encompassing vascular injury, chronic inflammation, and metabolic stress—creates a permissive environment for neurodegeneration. Neurodegenerative diseases represent accelerated, aberrant pathological processes occurring in specific brain regions against an aging backdrop. Structurally, brain aging drives generalized atrophy affecting the hippocampus, prefrontal cortex, and both gray and white matter (Aguilar-Hernández et al., 2023). Neurodegenerative conditions exacerbate this atrophy in disease-specific regions (e.g., hippocampus/entorhinal cortex in AD; substantia nigra pars compacta in PD) and compromise white matter integrity through demyelination and axonal damage (Groh and Simons, 2025). Cellular senescence represents a stable cell cycle arrest state induced by diverse stressors—including telomere shortening, DNA damage, oncogene activation, and oxidative stress (Melo Dos Santos et al., 2024). Critically, these cells are a primary source of sustained neuroinflammation (Andronie-Cioara et al., 2023), which serves as a key driver of neurodegenerative pathological cascades.

Organelle dysfunction, notably mitochondrial impairment, underlies cellular senescence and drives neuronal death in neurodegenerative diseases (Li et al., 2024b). Mitochondria, which serve as cellular powerhouses and a major source of ROS, exhibit multiple age-related alterations including reduced biogenesis, diminished clearance of damaged mitochondria (mitophagy) (Banarase et al., 2023), impaired electron transport chain function (Gasmi et al., 2024), increased ROS production, and attenuated antioxidant defense. Mitochondrial failure represents an early central event in nearly all neurodegenerative disorders. Key disease-associated proteins—such as PINK1 and Parkin in PD, as well as Amyloid Beta Precursor Protein and presenilin in AD—directly or indirectly regulate mitochondrial function or mitophagy (Strope and Wilkins, 2024). Accumulated misfolded proteins (e.g., Aβ precursor, PrP, mHTT) or environmental stressors potently activate the UPR. Sustained, unresolved ER stress triggers apoptotic pathways that execute neuronal death—a critical node in neurodegenerative pathogenesis (Ekundayo et al., 2024). Concurrently, autophagic-lysosomal impairment compromises clearance of misfolded proteins and damaged organelles, establishing it as a hallmark feature of neurodegeneration (Wu et al., 2025).

In summary, the interplay between aging and neurodegeneration constitutes a multilevel, interconnected vicious cycle. Aging provides the foundation: inherent processes—including structural/functional brain changes, accumulation of senescent cells, and systemic organellar decline—compromise cerebral resilience, undermining homeostatic integrity. Cellular senescence acts as an amplifier: senescent cells drive sustained neuroinflammation, tissue damage, and microenvironment deterioration, directly accelerating neurodegenerative pathology. Organellar dysfunction serves as the effector: functional decline in mitochondria, lysosomes/autophagy systems, and endoplasmic reticulum directly induces energy crises, oxidative stress, proteotoxicity, and calcium dysregulation, ultimately executing neuronal dysfunction and death programs. These deficits represent both consequences of aging and direct targets of neurodegenerative pathological proteins. Neurodegenerative diseases manifest as accelerated, pathological aging: triggered by genetic/environmental factors against an aging-compromised background, they feature disease-specific proteins [amyloid-β (Aβ), tau, α-synuclein [α-syn]) causing severe organellar dysfunction (particularly in mitochondria/lysosomes) within vulnerable brain regions. This converges with aberrant SASP activation in senescent glia, culminating in selective neuronal loss and region-specific functional deficits—an aberrantly accelerated pathological trajectory.

### Alzheimer’s disease

AD is a widely prevalent neurodegenerative disorder, marked by the degeneration of cortical and hippocampal neurons, which ultimately leads to cognitive and behavioral impairments (Li and Jin, 2025; Tinu et al., 2025). The primary pathological hallmarks are extracellular plaques composed of Aβ peptides and intracellular neurofibrillary tangles formed by hyperphosphorylated aggregates of Tau protein, a microtubule-associated protein found at elevated levels in the brains of AD patients. Aβ is generated through proteolytic cleavage of amyloid-beta precursor protein (APP) by two proteases termed β-secretase and γ-secretase. Presenilin 1 (PS1) and Presenilin 2 (PS2) (also known as PSEN1 and PSEN2, respectively) constitute the catalytic subunits of the γ-secretase complex. Mutations in genes encoding presenilin proteins and APP are responsible for certain familial forms of AD (Area-Gomez et al., 2018).

Mitophagy deficiency is mechanistically linked to AD progression. A recent study has found that inhibition of MAM-related autophagy is associated with AD (Yu et al., 2025). In APP/PSEN1 mice and Aβ-treated N2a/BV2 cells, significant suppression of the FUNDC1-Unc-51-like autophagy activating kinase 1 activation pathway in cortical and hippocampal regions correlates with reduced Beclin-1, LC3-II/LC3-I ratio, and p62 degradation, indicating impaired autophagic flux (Wang and Jia, 2023b). Pharmacological activation of the AMPK/mTOR/ULK1 axis enhances autophagy, attenuates Aβ burden and neuroinflammation, suppresses apoptosis, and improves cognitive function (Wang and Jia, 2023b). Conversely, Beclin 1 deletion in APP/PSEN1 microglia exacerbates autophagic failure, triggering NLRP3 inflammasome activation and pro-inflammatory cytokine release (Houtman et al., 2019). Beclin 1 overexpression in AD models restores mitophagic clearance, promotes microglial M2-polarization, and accelerates Aβ degradation through reactivated autophagic flux (Yang et al., 2023).

Several lines of evidence suggest that MAMs play a key role in the pathogenesis of AD. First, associated with MAMs, including VAPB, PTPIP51, IP3R1, MFN1, Mitofusin 2 (Sathyamurthy et al., 2024), phosphofurin acidic cluster sorting protein 2, and SigmaR1, have been observed in postmortem AD brain tissues, exhibiting either increases or decreases (Mao et al., 2022a). Furthermore, disruptions in ER-mitochondria contacts are evident in human postmortem AD tissues, even during early disease stages (Leal et al., 2020). Second, MAMs serve as platforms for key proteins implicated in AD pathogenesis: APP, Presenilin-1, and PS2 are all localized to MAMs. Third, following β-secretase cleavage of full-length APP, the resulting intermediate fragment (termed C99) is transported to MAMs and processed by γ-secretase to generate two peptides—the amyloid precursor protein intracellular domain and Aβ. This establishes MAMs as critical sites for Aβ biogenesis (Schreiner et al., 2015), the stabilization of MAM regulates the production of Aβ in AD (Zellmer et al., 2025). Finally, the E4 allele of apolipoprotein E, the primary genetic risk factor for AD, has been shown to modulate MAMs functionality (Agrawal et al., 2020).

A current study presents conflicting conclusions regarding the role of PS2 in regulating MAMs (Rossini et al., 2021). While one investigation reports that PS2 plays a pivotal role in this process, findings from SH-SY5Y cells and primary neuronal cultures indicate that PS1 is dispensable (Zampese et al., 2011). In contrast, a study using genetically engineered H4 glioblastoma cell lines expressing inducible mutant forms show that PS1 indeed modulates endoplasmic reticulum–mitochondria interactions (Han et al., 2021). This discrepancy may be attributed to model-specific variations, and the underlying mechanisms require further investigation.

Intriguingly, AD-associated PS2 mutants reduce mitochondrial Ca^2+^ uptake while increasing ER-mitochondria contact sites (Filadi et al., 2016), suggesting that enhanced ER-mitochondria connectivity may serve as a compensatory mechanism to facilitate Ca^2+^ transfer from the ER to mitochondria. The underlying mechanism involves the direct interaction of MAM-localized presenilins (PSs) with IP3Rs, where mutant forms of PS1 and PS2 can stimulate IP3R channel opening to mediate ER Ca^2+^ efflux (Johri and Chandra, 2021). These coordinated actions collectively facilitate enhanced Ca^2+^ release from the ER. In AD patients, elevated levels of MAM-associated proteins such as PACS2 and Sig-1R have been documented. PACS2, localized in the cytoplasm, influences mitochondria-endoplasmic reticulum contact sites by regulating the coupling between the ER and mitochondria (Dentoni et al., 2022). Sig-1R critically regulates AD pathogenesis by maintaining the structural integrity of MAMs, primarily through its molecular anchoring to IP3Rs and VDACs. Postmortem analyses of AD brains reveal elevated expression of IP3R and VDAC proteins. Pathologically, Sig-1R-mediated stabilization of MAM domains potentiates interactions between phosphorylated tau, Aβ oligomers, and γ-secretase, enhancing their proteotoxicity and culminating in mitochondrial dysfunction and caspase-dependent neuronal death during AD progression (Resende et al., 2022).

**Figure 7:**
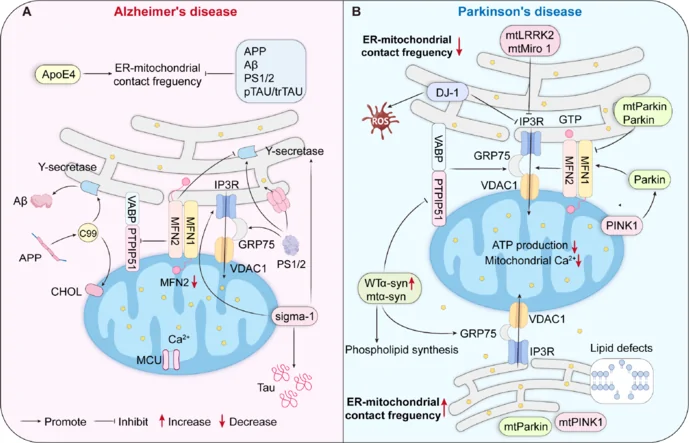
Interaction between aging and neurodegenerative diseases. (A) AD is characterized by the presence of Aβ plaques and tau tangles. MAMs play a crucial role in this process, as key AD-related proteins, including APP, PS1, and PS2, are localized to MAMs, where Aβ is generated. ApoE4 has also been shown to regulate MAM function. PS1 and PS2 influence the structural stability of the IP3 receptor–Grp75–VDAC1 complex and modulate γ-secretase activity, thereby affecting Aβ production. Although ER-mitochondria coupling is generally reduced in AD, the upregulation of MAM-associated proteins, along with Aβ-induced MAM formation, enhances calcium flow from the ER to mitochondria. This may represent a compensatory mechanism to restore mitochondrial calcium homeostasis. The sigma-1 receptor plays a multifaceted role by not only directly modulating calcium channels but also influencing pathological proteins in AD while stabilizing ER-mitochondria contact sites. (B) PD involves the degeneration of dopaminergic neurons and the aggregation of α-syn. The impact of α-syn on ER-mitochondria contacts remains inconsistent across studies; some evidence suggests that α-syn may facilitate calcium transfer by modulating calcium channels, potentially through its influence on ER-mitochondria contact sites. PINK1 and Parkin, which are closely associated with mitophagy, localize to MAMs and regulate ER-mitochondria signaling, although their precise mechanisms of action are not yet fully elucidated. DJ-1, also localized to MAMs, promotes ER-mitochondria contacts and calcium transfer. Its loss leads to excessive mitophagy and neuronal degeneration. AD: Alzheimer’s disease; ApoE4: apolipoprotein E4; APP: amyloid-beta precursor protein; Aβ: amyloid-beta; α-syn: alpha-synuclein; DJ-1: parkinsonism associated protein 7; ER: endoplasmic reticulum; MAMs: mitochondria-associated endoplasmic reticulum membranes; PINK1: PTEN-induced putative kinase 1; PS1: presenilin 1; PS2: presenilin 2; VDAC1: voltage-dependent anion channel 1.

The relationship between Aβ/APP and endoplasmic reticulum-mitochondria interactions shows inconsistent findings across studies, with some studies indicating increased contacts (Choi et al., 2023) and others reporting decreased associations (Fernandes et al., 2023). Moreover, the expression of disease-associated phosphorylated and truncated Tau elevates the number of ER-mitochondria contact sites (Mahakud et al., 2022). Analogous to PS2 mutants, truncated Tau has been reported to reduce basal ER Ca^2+^ levels. Conversely, one study found that impaired mitochondrial calcium efflux also contributes to the progression of AD, with further mechanisms potentially associated with the involvement of misfolded tau and β-amyloid proteins (Jadiya et al., 2019). Consequently, the subsequent increase in ER-mitochondria contacts may serve to restore mitochondrial Ca2+ homeostasis (**Figure 7**).

### Parkinson’s disease

PD is a prevalent neurodegenerative disorder characterized by degeneration of dopaminergic neurons in the substantia nigra and aggregation of α-syn into Lewy bodies (Espay and Lees, 2024). A previous study has reported that modulation of α-syn expression impacts ER-mitochondria signaling and associated functions such as Ca^2+^ homeostasis (Ramezani et al., 2023). However, no definitive consensus exists regarding the effects of α-syn on ER-mitochondria contacts. Initially, α-syn overexpression was found to increase MAM contacts and mitochondrial Ca^2+^ levels in HeLa cells (Calì et al., 2019).

A previous study demonstrates that MAM-localized α-syn binds to VAPB and disrupts its interaction with PTPIP51, thereby reducing ER-mitochondria contacts, and directly destabilizes ER-mitochondria tethering (Mori et al., 2022). Furthermore, loss-of-function mutations in PINK1 and Parkin (an E3 ubiquitin ligase; gene symbol PRKN), two proteins critical for mitophagy, represent established causative factors for PD (Berenguer-Escuder et al., 2019). McLelland et al. (2018) demonstrated that PINK1- and Parkin-mediated phospho-ubiquitination of Mitofusin 2 complexes promotes their degradation, resulting in the loss of ER-mitochondria contact sites and enhanced mitophagy. Conversely, another group reported that reduced Parkin or PINK1 expression similarly results in ER-mitochondria contact disengagement, which correlates with dysregulated Ca^2+^ homeostasis (Parrado-Fernández et al., 2018). Thus, while PINK1 and Parkin clearly play significant roles in ER-mitochondria signaling, their precise mechanistic contributions remain incompletely characterized.

DJ-1 (PARK7), a protein associated with PD, localizes to MAMs and facilitates the formation of ER-mitochondria contacts (Fan et al., 2025). DJ-1 interacts with the IP3R-GRP75-VDAC Ca^2+^ channel complex within MAMs to enhance MAM-mediated Ca^2+^ transfer. Concurrently, DJ-1 functions as a deglycase and a small antioxidant protein, playing a critical role in scavenging ROS, which is mechanistically linked to PD pathogenesis (Fan et al., 2025). Loss-of-function mutations or deletions in the PARK7 gene, which encodes DJ-1, are causative for early-onset forms of PD (Repici and Giorgini, 2019). Although DJ-1 primarily resides in the cytoplasm, it dynamically accumulates at ER-mitochondria contact sites, where it stabilizes membrane tethering. Consequently, DJ-1 deficiency in PD leads to excessive mitophagy, impairs neuronal energy supply through mitochondrial dysfunction, and ultimately precipitates the degeneration of dopaminergic neurons (Skou et al., 2024; **Figure 7**).

### Frontotemporal dementia and amyotrophic lateral sclerosis

ALS and FTD represent two clinically interrelated and currently incurable neurodegenerative disorders. ALS is primarily characterized by the degeneration of both upper and lower motor neurons. FTD encompasses a spectrum of neurodegenerative conditions clinically defined by behavioral disturbances and progressive neuronal loss in the frontal and temporal lobes, resulting in characteristic cortical atrophy. The pathological hallmarks of ALS/FTD primarily involve aberrant inclusions of TDP-43 (Rummens and Da Cruz, 2025) and fused in sarcoma (FUS) protein (Vazquez-Sanchez et al., 2024). Emerging evidence indicates that disrupted MAMs constitute a common pathological feature in ALS. MAMs dysfunction inactivates TANK-binding kinase 1 and exacerbates proteostatic stress, leading to increased cellular vulnerability *in vitro* and motor deficits *in vivo* (Watanabe et al., 2023b). Mechanistically, TDP-43 and FUS impair mitochondrial-ER interactions by suppressing the VAPB-PTPIP51 complex through the activation of glycogen synthase kinase 3β (GSK3β), thereby disrupting Ca^2+^ flux regulation between these organelles (Markovinovic et al., 2024).

Repeat expansions in the *C9ORF72* gene represent a frequent genetic cause of both ALS and FTD. Mitochondrial dysfunction and chronic ERS are well-established contributors to C9ORF72-linked disease pathogenesis. Induced pluripotent stem cell (iPSC)-derived motor neurons harboring the C9ORF72 hexanucleotide repeat expansion exhibit diminished mitochondrial membrane potential, heightened ERS, and reduced levels of Bcl-2—an anti-apoptotic protein critical for modulating cellular Ca^2+^ signaling (Dafinca et al., 2016). Concurrently, increased expression of glucose-regulated protein 78 (GRP78; also known as BiP), a hallmark of the ERS response, is observed alongside ultrastructural mitochondrial swelling. These cells display disrupted Ca^2+^ homeostasis, characterized by augmented Ca^2+^ release upon depolarization, delayed Ca^2+^ recovery post-glutamate stimulation, and diminished levels of calbindin, a cytosolic Ca^2+^-buffering protein, collectively impairing excess Ca^2+^ clearance. Furthermore, mirroring the phenotype of TARDBP-mutant iPSC-derived motor neurons, Ca^2+^-permeable subunits of glutamatergic AMPA and NMDA receptors are upregulated, while mitochondrial Ca^2+^ buffering capacity is suppressed (Dafinca et al., 2020). This molecular configuration exacerbates glutamate excitotoxicity, a pathological hallmark of ALS in motor neurons, by compromising mitochondrial Ca^2+^ uptake (Quessada et al., 2021). *In vivo*, mitochondrial abnormalities are induced by poly-GR, a dipeptide repeat protein inclusion pathognomonic of C9ORF72-ALS. In murine overexpression models, poly-GR directly binds mitochondrial ATP synthase subunit ATP5A1 (ATP5F1A), triggering its ubiquitination and subsequent proteasomal degradation, consistent with the reduced ATP5A1 levels detected in patient-derived brain tissues (Choi et al., 2019). Notably, these poly-GR-associated mitochondrial defects manifest prior to the emergence of other cellular pathologies, underscoring mitochondrial dysfunction as an early pathogenic driver in C9ORF72-ALS (Burley et al., 2022).

Mutant SOD1 accumulates at MAMs, particularly in neurons, a phenomenon attributed to its aberrant binding to mitochondrial membranes where it suppresses ER-mitochondria tethering (Sakai et al., 2021). This pathogenic accumulation drives reduced ATP production, dysregulated intracellular Ca^2+^ homeostasis, and depletion of MAM-resident proteins including SigmaR1, IP3R3, and calnexin, indicative of structural and functional MAM compromiseand mitochondrial dysfunction (Wang et al., 2025b). Concurrently, mutant SOD1 induces conformational changes in Bcl-2 that impair VDAC1-mediated Ca^2+^ shuttling. Such perturbations inhibit mitochondrial uptake of Ca^2+^ and ADP, culminating in diminished ATP synthesis and exacerbated oxidative stress. These cascading deficits ultimately trigger mitochondrial depolarization, cellular injury, and neuronal death (Watanabe et al., 2016).

**Figure 8:**
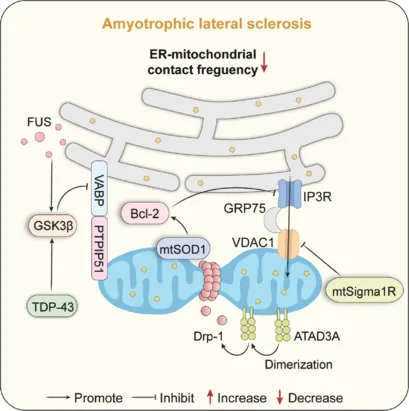
Frontotemporal dementia and amyotrophic lateral sclerosis (FTD/ALS). ALS features motor neuron degeneration with TDP 43 and FUS inclusions. MAMs dysfunction is common. TDP-43 and FUS disrupt VAPB- PTPIP51 complex, impairing Ca^2+^ flux. Mutant SOD1 accumulates at MAMs, reducing ATP production and disrupting Ca^2+^ homeostasis. Sig-1R, crucial in ALS, forms complexes with ATAD3A to regulate MAM assembly and mitochondrial fission, maintaining Ca^2+^ regulation. ATAD3A: ATPase family AAA domain containing 3A; Bcl-2: B-cell lymphoma-2; VDAC1: Ca^2+^: calcium ion; Drp1: dynamin-related protein 1; FUS: fused in sarcoma; IP3R: inositol 1, 4, 5-trisphosphate receptor;MAMs: mitochondria-associated endoplasmic reticulum membranes; PTPIP51: protein tyrosine phosphatase interacting protein 51; Sig-1R: sigma-1 receptor; SOD1: superoxide dismutase 1; TDP-43: TAR DNA-binding protein 43; VAPB: vesicle-associated membrane protein associated protein B; voltage-dependent anion channel 1.

Sig-1R, a MAM-specific factor highly expressed in spinal motor neurons, plays a critical role in ALS, as its mutations are implicated in ALS, ALS/FTD, and juvenile ALS (ALS16) (Sharma et al., 2021). Under physiological conditions, Sig-1R activation alleviates ERS and mitochondrial calcium overload through the PERK and IP3R-VDAC1-MCU signaling pathway (Li et al., 2025c). Sig-1R mutations drive ALS pathogenesis through impaired binding of mutant proteins to inositol IP3R3, resulting in ER-mitochondria dissociation, disrupted Ca^2+^ homeostasis, and diminished ATP synthesis (Watanabe et al., 2016). Furthermore, Sig-1R deficiency decreases MAM-resident proteins involved in Ca^2+^ regulation, such as IP3R3. A previous study indicates that the formation of MAM may depend on ATPase family AAA domain-containing protein 3A (ATAD3A), with Sig-1R potentially playing a regulatory role in this process (Watanabe et al., 2023a). In ALS, Sig-1R interacts with ATAD3A to form a complex that promotes MAM assembly and suppresses mitochondrial fission. Conversely, Sig-1R depletion increases mitochondrial fission and MAM loss (Watanabe et al., 2023a). ATAD3A homodimerization recruits Drp1 to drive mitochondrial fission, whereas the Sig-1R–ATAD3A complex inhibits ATAD3A dimerization, facilitating mitochondrial elongation and MAMs stabilization—a mechanism critical for rectifying Ca^2+^ dysregulation in ALS (Zhao et al., 2019a; **Figure 8**).

In summary, Sig-1R and ATAD3A serve as pivotal regulators of MAM integrity to maintain mitochondrial homeostasis in neurological disorders. Therapeutic targeting of the Sig-1R–ATAD3A complex at MAMs may offer a novel strategy to counteract mitochondrial dysfunction in neurodegenerative diseases.

### Multiple sclerosis

MS is a complex inflammatory and neurodegenerative disorder characterized by autoimmune-driven demyelination in the central nervous system (CNS), where aberrant activation of autoreactive T lymphocytes initiates pathogenesis (López-Muguruza and Matute, 2023). These cells orchestrate disease progression through CNS infiltration, release of pro-inflammatory cytokines, and macrophage activation, thereby amplifying neuroinflammation (Kirschner et al., 2025). Lymphocyte activation involves dysregulated signaling pathways, with mitochondrial dysfunction (Pukoli and Vécsei, 2023) and oxidative stress (Gülow et al., 2024) serving as critical modulators of disease mechanisms. Conversely, impaired apoptosis compromises the elimination of pathogenic autoreactive cells, exacerbating immune-mediated tissue damage (Zhou et al., 2023). While the structural and functional integrity of MAMs and their Ca^2+^ handling dynamics remain underexplored in multiple sclerosis, convergent indirect evidence implicates MAM dysregulation in the disease pathogenesis.

Postmortem analyses of tissues from MS patients and MS animal models demonstrate widespread dysregulation of mitochondrial function in neurons and oligodendrocytes (Pukoli and Vécsei, 2023). Studies in animal models show that disruption of mitochondrial homeostasis, restricted to axons, occurs during the asymptomatic phase in a murine model of MS (Buonvicino et al., 2023). Peripheral immune cells in MS patients, particularly CD4^+^ T cells, exhibit aberrant mitochondrial phenotypes and/or deficient oxidative phosphorylation. In experimental models, this dysregulation exacerbates inflammation mediated by CNS autoimmunity (Cortes-Figueiredo et al., 2024). These defects drive metabolic reprogramming, which manifests as elevated glycolytic flux—though paradoxically reduced in one study—along with increased activity of glycolytic enzymes (das Neves et al., 2023).

**Figure 9:**
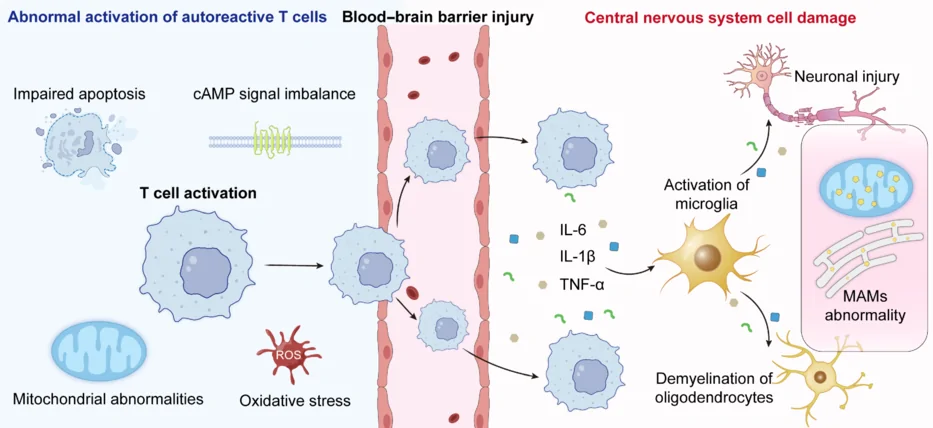
Pathological mechanisms of multiple sclerosis. The pathological mechanisms of MS involve several interconnected processes. Abnormal activation of autoreactive T cells, driven by factors such as impaired apoptosis and cAMP signaling imbalance, contributes to blood-brain barrier injury, allowing T cells to infiltrate the central nervous system. This infiltration triggers the release of pro-inflammatory factors (IL-6, IL-1β, and TNF-α), which activate microglia. As a result, this cascade leads to damage of central nervous system cells, including neuronal injury, oligodendrocyte demyelination, and abnormalities in MAMs, illustrating the multi-step pathological cascade of multiple sclerosis. cAMP: Cyclic adenosine monophosphate; IL-1β: interleukin-1 beta; IL-6: interleukin-6; MAMs: mitochondria-associated endoplasmic reticulum membranes; MS: multiple sclerosis; TNF-α: tumor necrosis factor-alpha.

Autophagy represents a double-edged sword in the pathogenesis of MS. Heightened autophagic flux may exacerbate neuropathology by potentiating autoimmune responses, while mitochondrial dysfunction in MS induces compensatory expression of autophagy-related genes (Liang and Le, 2015). This duality positions autophagy as a context-dependent modulator with both protective and deleterious effects. A case-control study revealed significant dysregulation of autophagy-related genes in MS patients compared to controls, collectively indicating autophagic dysfunction that may compromise cellular homeostasis (Igci et al., 2016). Autophagy exerts neuroprotective effects in MS by scavenging ROS, thereby mitigating disease pathogenesis and progression (Righes et al., 2025). Paradoxically, heightened autophagic flux may also potentiate neuropathology through autoimmune enhancement. MS patients exhibit elevated oxidative damage to phospholipids and DNA, along with increased oxidative stress compared to healthy controls. Notably, diminished autophagic activity correlates with impaired ROS clearance and exacerbated inflammation. Furthermore, defective autophagy compromises the clearance of misfolded proteins and damaged neurons (Igci et al., 2016). Mitochondrial dysfunction in MS induces both mitophagy and general autophagy activation. Paradoxically, pharmacological inhibition of these processes enhances oligodendrocyte-mediated remyelination and improves axonal myelination. Notably, the antipsychotic agents clozapine and haloperidol mitigate cuprizone-induced motor dysfunction in murine models through the suppression of mitophagic and autophagic flux (Patergnani et al., 2021; **Figure 9**).

Thus, although the mechanistic role of MAMs in MS pathogenesis remains incompletely defined, multiple pathological processes implicated in MS exhibit intimate pathophysiological convergence with MAM functionality. Consequently, structural and functional interrogation of MAM domains represents a promising frontier for future therapeutic targeting.

## Clinical Drug Trials and Therapeutic Safety

MAMs constitute dynamic membrane contact sites between the ER and mitochondria, critically regulating neuronal survival through coordinated Ca^2+^ shuttling, lipid metabolism, and bioenergetic homeostasis. In aging and neurodegenerative pathologies, including AD, PD, and ALS, MAM dysregulation disrupts Ca^2+^ homeostasis, triggering mitochondrial damage, oxidative stress, and apoptotic cascades. While direct MAM-targeting therapeutics remain predominantly in early-stage development, approved agents that indirectly modulate Ca^2+^ dynamics (e.g., anti-amyloid antibodies) exhibit ambiguous mechanistic associations with MAM functionality. Although research on the structure and function of MAMs is increasingly abundant, drug development targeting key MAM components remains significantly challenging due to their unique physiological mechanisms within cells (**Additional Table 1**).

**Additional Table 1:** Key targets of mitochondria-associated endoplasmic reticulum membranes in neurodegenerative disorders: mechanisms and druggability challenges

Target	Core function	Undruggability challenges
IP3R	Endoplasmic reticulum calcium release channel; regulates ER-mitochondrial calcium flux	High conformational dynamics; cryptic small-molecule binding pockets
VDAC	Mitochondrial outer membrane calcium transport hub	Systemic toxicity risk upon direct inhibition (e. g., cardiotoxicity) due to ubiquitous calcium homeostasis roles
MFN1/2	Regulates mitochondrial fusion and ER-mitochondria tethering	Requires protein-protein interaction (PPI) disruption; lacks efficient organelle-specific delivery systems
MCU	Core mitochondrial calcium uniporter channel	Global calcium signaling modulation; limited tissue-specific delivery strategies
Sig-1R	Stabilizes ER-mitochondria contacts; modulates calcium homeostasis and stress response	Receptor pleiotropy (calcium signaling/neurotransmitter regulation) complicates efficacy attribution

IP3R: Inositol 1,4,5-trisphosphate receptor; MCU: mitochondrial calcium uniporter; MFN1: mitofusin 1; MFN2: mitofusin2; Sig-1R: sigma-1 receptor; VDAC: voltage-dependent anion channel.

### Direct modulators of calcium channels

Pharmacological modulators targeting calcium channels are under active development and optimization. Reported regulatory molecules likely influence cellular functions through diverse biochemical pathways.

Xestospongins (XeA, XeB, XeC, XeD): The xestospongins, macrocyclic bis-1-oxaquinolizidine alkaloids first isolated from the marine sponge Xestospongia, have been established as cell-permeable pharmacological antagonists of IP3R-evoked Ca^2+^ mobilization (Gambardella et al., 2021). Emerging research has shifted toward investigating xestospongins in neurodegenerative contexts, revealing potent neuroprotective effects. Notably, xestospongin administration attenuates cognitive deficits and ameliorates neuropathological hallmarks in APP/PS1 AD mouse models (Wang et al., 2019).

Sephin1: Sephin1, a phosphatase inhibitor and structural analog of the central antihypertensive agent guanabenz (an α2-adrenergic receptor agonist), prevents the dephosphorylation of eIF2α, thereby attenuating proteotoxic stress responses induced by protein misfolding (Crespillo-Casado et al., 2018). This compound may exert indirect modulatory effects through the putative stabilization of IP₃R. Current research has demonstrated its therapeutic potential in multiple sclerosis models (Chen et al., 2019), while clinical trials for ALS are actively underway.

VDAC1: VDAC1 critically contributes to the pathogenesis of AD through dysregulated expression and functional modulation. Pharmacological inhibitors of anion transport—including DIDS, H_2_DIDS, DNDS, SITS, and DPC—directly interact with VDAC1 to reduce channel conductance, thereby suppressing apoptosis triggered by ROS overproduction, cytosolic Ca^2+^ overload, mitochondrial membrane potential (ΔΨm) dissipation, and pathological VDAC1 oligomerization (Ben-Hail and Shoshan-Barmatz, 2016). VDAC1 contains specific Ca^2+^-binding domains, enabling its channel activity to be modulated by divalent metal ions.

VBIT-4: VBIT-4, a VDAC1-specific inhibitor identified through compound screening by the Shoshan-Barmatz laboratory, directly interacts with VDAC1 to reduce its channel conductance (Ben-Hail and Shoshan-Barmatz, 2016). Experimentally, VBIT-4 inhibits VDAC1 oligomerization and Cyt c release across multiple cell lines, demonstrating protective effects against apoptosis induced by mitochondrial dysfunction.

VDAC1 N-terminal peptide: Separately, a non-penetrating VDAC1 N-terminal peptide (VDAC1-N-Ter peptide) was designed based on sequence analysis of the VDAC1 N-terminus and Aβ. This peptide binds to Aβ and inhibits its toxicity (Smilansky et al., 2015).

The MCU, the central channel regulating mitochondrial Ca^2+^ uptake, is critically involved in cellular energy metabolism, mitophagy, and apoptosis. Dysfunction of MCU leading to mitochondrial calcium overload is closely associated with the pathogenesis of neurodegenerative diseases, including AD, PD, and ALS. MCU dysregulation can trigger mitochondrial impairment, excessive ROS production, activation of apoptotic signaling, and synaptic damage, ultimately promoting neuronal death. Although MCU represents a promising therapeutic target, no drugs directly targeting it have advanced to clinical trials for neurodegenerative disorders. Current MCU inhibitors, primarily used as research tools, face clinical translation challenges due to limitations in selectivity, permeability, or safety. Key inhibitors include ruthenium-based compounds such as ruthenium red (RuRed), Ru360, and Ru265 (Rodríguez-Prados et al., 2023), as well as small molecules like mitoxantrone and DS16570511 (Belosludtsev et al., 2021). A recent groundbreaking study has revealed for the first time that berberine (also known as Huanglian Su or Berberine), an active component of the Chinese herb *Coptis chinensis* (Huanglian), acts as a novel MCU inhibitor. It significantly suppresses mitochondrial Ca^2+^ uptake by disrupting the assembly of the MCU-EMRE complex. Mechanistically, berberine directly binds to the juxtamembrane loop (JML) domain of MCU—a critical regulatory region for MCU-EMRE complex assembly and channel gating—to exert its inhibitory effect. Notably, unlike conventional MCU inhibitors that primarily target the Ca^2+^-binding site, berberine induces conformational changes in MCU, specifically destabilizing the MCU-EMRE complex and ultimately leading to channel closure and prevention of Ca^2+^ influx (Zhao et al., 2025). Given the risk of cardiac or skeletal muscle dysfunction from systemic MCU inhibition due to its essential role in energy metabolism, indirect strategies targeting upstream regulators have emerged. For example, inhibiting the plasma membrane calcium channel Orai1 – which promotes MCU expression via CREB phosphorylation – reduces MCU levels and demonstrates neuroprotective effects (Luo et al., 2024).

Tacedinaline, a class-selective HDAC inhibitor, counters Aβ-induced neuronal dysfunction in AD by reducing Aβ levels. It achieves this by modulating the transcriptional activity of Ca^2+^-handling proteins at MAMs, thereby suppressing abnormal ER-Ca^2+^ retention and mitochondrial Ca^2+^ accumulation mediated by the IP3R-GRP75-VDAC channel complex (Marinho et al., 2023).

### Pharmacological modulation of sigma-1 receptor

Sig-1R, a molecular chaperone predominantly localized at the MAMs, plays a central protective role in neurodegenerative diseases by regulating key processes including calcium homeostasis, mitochondrial function, ERS, and neuroinflammation. Specifically, Sig-1R enhances calcium transfer between the ER and mitochondria, thereby optimizing mitochondrial ATP production, suppressing pathological calcium overload, and modulating neuroinflammatory and immune responses. Research demonstrates that Sig-1R agonists exhibit potent anti-amnesic and neuroprotective effects across various animal models of cognitive impairment.

For instance, pridopidine, acting via Sig-1R, stabilizes mushroom spines in AD mouse models (Ryskamp et al., 2019) and also shows therapeutic potential in models of Parkinson’s disease (Drewes et al., 2025) and ALS. Similarly, the Sig-1R agonist fluvoxamine significantly reduces Aβproduction in cellular models of Aβ deposition by inhibiting γ-secretase activity. Importantly, Sig-1R itself exerts a significant influence on γ-secretase activity and Aβ generation, contributing to its protective effects on cognitive function in mice. SA4503 (Cutamesine) is a potent and selective Sig-1R agonist (Mercer et al., 2024). Research has demonstrated its ability to enhance neurotrophic factor release, promote neuronal repair, and reduce neuronal apoptosis (Tanji et al., 2021).

Blarcamesine (ANAVEX2-73), an orally administered small-molecule agonist targeting Sig-1R, modulates both Sig-1R and muscarinic receptors and represents a promising clinical candidate. It effectively enhances mitochondrial activation in neurons while mitigating neuroinflammation (Villard et al., 2011). Preclinical studies demonstrate its beneficial effects on mitochondrial dysfunction and neuroinflammation, indicating disease-modifying potential to halt and/or reverse AD progression (Maurice, 2025). Animal models reveal additional anticonvulsant, anti-amnesic, neuroprotective, and antidepressant properties, suggesting therapeutic utility for other central nervous system disorders, including epilepsy. Clinical development includes completed Phase 2a and 2b/3 trials for AD, as well as a Phase 2 proof-of-concept study for PD dementia (Hampel et al., 2020). In December 2022, a Phase IIb/III trial involving 509 patients met both primary and key secondary endpoints: over 84% of treated patients exhibited a clinically meaningful reduction (≥0.50 points) in ADAS-Cog scores compared to placebo, with responders showing an average decline of 4.03 points (Macfarlane et al., 2025). Blarcamesine increased the probability of functional improvement by 167% (≥ 3.5-point increase in ADCS-ADL), demonstrating significant clinical benefits in AD patients (Macfarlane et al., 2025). Furthermore, it significantly reduced cognitive decline by 45% and achieved secondary endpoints with a 0.42-point reduction in clinical cognitive and functional decline (Macfarlane et al., 2025).

Furthermore, several compounds that act as agonists or antagonists of the Sig-1R exhibit therapeutic potential for neurodegenerative diseases, although their development remains at the preclinical stage. PRE-084, a selective Sig-1R agonist, mitigates neuroinflammation and oxidative stress in experimental models (Shi et al., 2025) and demonstrates neuroprotective effects in SOD1-mutated ALS mouse models (Peviani et al., 2014).

### Pharmacological modulation of endoplasmic reticulum-mitochondria tethering

MFN1 and Mitofusin 2, localized on the mitochondrial outer membrane, mediate mitochondrial fusion to maintain mitochondrial network integrity while concurrently facilitating endoplasmic reticulum-mitochondria tethering. These proteins coordinate energy metabolism, regulate calcium homeostasis, and respond to oxidative stress. The VAPB-PTPIP51 complex serves as a core molecular regulator of ER-mitochondria signaling, forming critical physical linkages within MAMs to govern calcium flux, lipid metabolism, and autophagy. In neurodegenerative diseases, disruption of these connections induces mitochondrial dysfunction, oxidative stress, and neuronal apoptosis.

S89 is a small-molecule agonist that specifically promotes mitochondrial fusion by activating endogenous MFN1 (Guo et al., 2023). It directly interacts with a loop region in the helix bundle 2 domain of MFN1, stimulating GTP hydrolysis and vesicle fusion. S89 rescues mitochondrial and cellular defects induced by mitochondrial DNA mutations, oxidative stress (paraquat), ferroptosis inducers (RSL3), or CMT2A-associated mutations through MFN1 potentiation (Guo et al., 2023). Although not yet validated in central nervous system neurodegeneration models, its selective activation of MFN1—without significant effects on the homologous protein Mitofusin 2—avoids the risks associated with hyperactivation (Guo et al., 2023), suggesting superior therapeutic potential.

Luteolin (LUT), a flavonoid polyphenol compound found ubiquitously in fruits, vegetables, flowers, and medicinal herbs (Zhu et al., 2024a), mitigates cognitive impairment in AD mouse models by suppressing ERS-dependent neuroinflammation (Kou et al., 2022). This neuroprotective effect may be mediated through enhanced ER-mitochondria contacts (Naia et al., 2021). Elamipretide, a mitochondria-targeted tetrapeptide, demonstrates therapeutic efficacy and safety in multiple mitochondrial disorders. In neurodegenerative contexts, studies have demonstrated its ability to potentiate mitochondrial respiratory capacity, enhance mitochondrial fusion, suppress fission, and stimulate mitophagy (Zhang et al., 2024). Furthermore, elamipretide attenuates neuroinflammation and oxidative stress while facilitating the clearance of neurotoxic proteins such as Aβ(Nhu et al., 2021).

Current research on therapeutic agents targeting ER-mitochondrial tethering remains scarce, with no candidates for neurodegenerative diseases having entered clinical trials. Consequently, pharmacological modulation of this inter-organelle interface represents a promising frontier for novel neuroprotective strategies.

## Current Status of Clinical Translation and Application

The relentless global burden of neurodegenerative disorders has catalyzed intensive research into subcellular organelles as therapeutic targets, with the MAM emerging as a critical signaling hub. While adult CNS neurons exhibit limited self-repair capacity, compelling evidence suggests that targeted pharmacological manipulation of MAMs, as key endoplasmic reticulum-mitochondria contact sites, enables neuronal regeneration, demonstrating substantial promise for therapeutic development in spinal cord injury and neurodegenerative disorders (Sathyamurthy et al., 2024). Despite decades of investigation, direct pharmacological modulation of MAM-resident proteins—including IP3R, VDAC, MCU, Mfn1/2, and Sig-1R—remains predominantly confined to preclinical or early-phase trials. This translational impasse reflects persistent biological and pharmacological challenges that demand innovative solutions.

IP3R and VDAC exemplify targets constrained by structural dynamics and systemic toxicity. The high conformational flexibility of IP3R renders its small-molecule binding sites cryptic, limiting the development of allosteric inhibitors despite their potential to normalize ER-mitochondrial calcium flux. VDAC inhibition, while theoretically beneficial for restoring mitochondrial calcium homeostasis, carries significant cardiotoxicity risks due to its ubiquitous role in cellular calcium signaling. Similarly, MCU modulators face challenges in achieving mitochondrial specificity, as global calcium signaling disruption may compromise neuronal excitability or cardiac function.

Mfn1/2 agonists, designed to enhance mitochondrial fusion and ER-mitochondrial tethering, struggle with delivery constraints. Current molecules lack organelle-specific targeting, resulting in off-tissue effects and subtherapeutic concentrations at neuronal MAM interfaces. Meanwhile, Sig-1R ligands confront pleiotropy: their simultaneous modulation of calcium signaling, chaperone activity, and neuroinflammation complicates efficacy attribution in clinical trials. Despite robust preclinical data, Blarcamesine (NCT04314934), currently the most promising drug for neurodegeneration (Degirmenci et al., 2023), failed to meet the primary endpoint, necessitating improvements in research protocols for future studies (Ette et al., 2025).

Notably, indirect therapeutic strategies show promise. Certain agents target upstream regulators; for example, Sephin1 (an eIF2α stabilizer) acts by prolonging eIF2α phosphorylation, thereby reducing proteotoxic stress and stabilizing IP₃R conformation. The company’s ongoing trial in ALS (NCT05508074) seeks to decouple calcium dysregulation from the protein misfolding cascade. Concurrently, Blarcamesine (Anavex®2-73), a Sig-1R agonist, underwent a protocol amendment following Phase II/III trials (NCT04314934). It currently demonstrates therapeutic effects across multiple disorders, and in 2024, the company announced the submission of a Marketing Authorization Application for Blarcamesine to the European Medicines Agency (**Additional Table 2**).

**Additional Table 2:** Active clinical trials targeting mitochondria-associated endoplasmic reticulum membranes (MAMs)-related pathways in neurodegenerative disorders (North America)

OmicalTrials. gov ID / Study name	Target/mechanism	Intervention	Indication	Phase	Current status
NCT05508074	IP3R stabilizer (eIF2α dephosphorylation inhibition)	Sephin1 (IFB-088)	Bulbar-onset ALS	Phase IIa	Completion 2025; demonstrates slowed functional decline
NCT04314934	Sigma-1 receptor agonist	Blarcamesine	Alzheimer's disease	Phase II/III	Endpoint not met (2024; protocol refinement ongoing
NCT06355531	Tau pathology modulation (O-GlcNAcase inhibitor)	FNP-223	Progressive supranuclear palsy	Phase II	Active; FDA Fast Track designation granted

This table summarizes the currently active clinical trials in North America targeting MAM-associated pathways in neurodegenerative diseases. MAMs serve as critical communication hubs between the endoplasmic reticulum and mitochondria, and their dysregulation is closely implicated in the pathological mechanisms of multiple neurodegenerative disorders, including dysregulated calcium signaling, mitochondrial dysfunction, and endoplasmic reticulum stress. ALS: Amyotrophic lateral sclerosis; FDA: U.S. Food and Drug Administration; IP3R: inositol 1,4,5-trisphosphate receptor; MAMs: mitochondria-associated endoplasmic reticulum membranes; eIF2α: eukaryotic initiation factor 2 alpha; O-GlcNAcase: O-linked N-acetylglucosaminidase.

Concurrently, novel diagnostic approaches exemplified by the Sigma-1 receptor PET tracer [^11^C]CNY-01—developed through a collaboration with Harvard University—are poised for regulatory approval by the National Institutes of Health (NIH). This advancement will enable non-invasive quantification of *in vivo* Sig-1R density, accelerating clinical translation (Bai et al., 2025). Gene and cell therapies represent an emerging class of treatments with distinct advantages over conventional approaches, demonstrating superior precision targeting and reduced costs. Consequently, regulatory agencies like the FDA often expedite their review processes. For instance, SNUG01 (a TRIM72 modulator), delivered via intrathecal administration of an rAAV9 vector encoding human TRIM72, received FDA orphan drug designation for ALS in 2025; this therapy restores mitochondrial function by enhancing membrane repair mechanisms. Similarly, XS-411 (iPSC-derived dopamine neurons) rapidly advanced for PD treatment by directly replenishing lost dopaminergic neurons to potentially restore dysfunctional neuronal circuitry. In clinical studies, patients exhibited a 40% improvement in motor scores (MDS-UPDRS) and a 60% reduction in “on-off” fluctuations, with no serious adverse events, leading to FDA “Special Protocol Assessment (SPA)” status in February 2025, which bypassed technical review bottlenecks. Concurrently, a dopaminergic neuron therapy from BlueRock Therapeutics received FDA accelerated Phase 3 approval in January 2025 based on demonstrated engraftment efficacy, bypassing Phase 2 requirements to become the first neural cell therapy to achieve this accelerated pathway (**Additional Table 3**).

**Additional Table 3:** FDA-related approval projects

FDA accelerated cell therapy approvals			
Drug/therapy	Developer	Regulatory action	Outcome impact
Dopaminergic neuron therapy	BlueRock Therapeutics	FDA granted Phase III acceleration (January 2025) following positive Phase I results	Skipped Phase II trial requirement
XS-411 iPSC-derived neurons	Shize Biotech	FDA regulatory flexibility provisions	Streamlined clinical protocols; reduced development costs
**FDA Special designations for MAMs-related neurodegenerative therapies**			
**Drug/therapy**	**Target/Mechanism**	**Indication**	**Regulatory progress**

FNP-223	Indirect tau pathology modulation (OGA inhibition)	Progressive supranuclear palsy	FDA Fast Track designation (2025)
SNUG01	TRIM72 (mitochondrial function restoration)	Amyotrophic lateral sclerosis	FDA Orphan Drug designation (2025)
IPG8294 (Immune Pharma)	NAD+ hydrolase inhibition (compensatory MCU functional restoration)	Mitochondrial myopathy	FDA IND clearance + Orphan status (2025)

This table summarizes the accelerated approval pathways and special designations granted by the U.S. FDA for neurodegenerative and mitochondria-related disorders, highlighting the regulatory support for innovative therapies in these areas. iPSC: Induced pluripotent stem cells; MAMs: mitochondria-associated endoplasmic reticulum membranes.

NIH-funded projects have predominantly focused on mechanistic studies of MAMs and associated calcium channel proteins over recent decades (**Additional Table 4**). This sustained research effort has yielded a substantial body of knowledge regarding MAM structure, function, and regulatory mechanisms. Consequently, future initiatives are shifting toward target-specific therapeutic development for clinical translation.

**Additional Table 4:** NIH-funded projects targeting MAMs mechanisms

Grant ID	Institution	Target	Core objectives
5R01NS038082-09	UT Southwestern medical center	IP3R	Structure and function of IP3R
5R01NS042319-06	Baylor college of medicine	VDAC	The Role of Mitochondrial VDACs in Apoptosis
5R03DA027182-02	Brown university	Sigma-1 receptor	Proteomic characterization of the sigma-1 receptor and its signaling complex
1R01HL165797-01	University of Utah	MCU	Regulation of the mitochondrial calcium uniporter

This table enumerates a series of basic research projects funded by the U.S. National Institutes of Health (NIH) that collectively focus on elucidating the core molecular mechanisms underlying the function of MAMs. IP3R: Inositol 1,4,5-trisphosphate receptor; MCU: mitochondrial calcium uniporter; MAMs: mitochondria-associated endoplasmic reticulum membranes; NIH: National Institutes of Health; VDAC: voltage-dependent anion channel.

## Limitations

This review summarizes the diverse roles of MAM-mediated Ca^2+^ signaling in neurodegenerative diseases; however, it has several limitations. We focused solely on five neurological disorders: AD, PD, ALS, MS, and FTD. Consequently, we cannot comprehensively delineate the functions of MAM-mediated Ca^2+^ signaling pathways across all neurological diseases. Furthermore, the conclusions drawn in this study are primarily based on cellular and transgenic animal models, which may not fully recapitulate the complexity of human diseases, particularly sporadic cases that involve multifactorial interactions and long-term chronic progression. Although experiments utilizing patient-derived cells, such as iPSC-differentiated neurons and postmortem tissues, have been incorporated, more in-depth exploration is required to systematically elucidate human-specific pathological features. Acquiring living human brain tissue for ultrastructural or functional analysis remains constrained by both ethical and technical barriers. The development of drugs specifically targeting the modulation of MAM integrity or calcium transport faces significant challenges. For instance, inhibiting the MCU may disrupt basal energy metabolism, while modulating calcium channels (e.g., IP3R, VDAC) could impact broad physiological processes. Most drugs mentioned in this article (such as xestospongins, VDAC inhibitors, and Sigma-1 receptor (Sig-1R) agonists) are currently in preclinical or early clinical stages. Their long-term safety, specificity for MAMs, and ability to cross the blood-brain barrier require thorough evaluation. Future research endeavors will demand substantial time and effort to further elucidate the involvement of MAM-mediated Ca^2+^ signaling pathways in pathogenesis, neurorepair, neuroregeneration, and the design of related targeted therapeutics for neurodegenerative diseases.

## Conclusion

This review highlights a crucial yet often neglected aspect of neurodegenerative disease pathology: the role of MAMs in mediating Ca^2+^ flux. By framing MAMs as a dynamic signaling hub, the review emphasizes their importance in facilitating inter-organelle communication, particularly in the context of Ca^2+^ transfer between the endoplasmic reticulum and mitochondria. Unlike previous studies that have focused on the role of MAM in specific diseases, this review uniquely posits that the integrity and function of MAMs may serve as a critical determinant of cellular outcomes during neural repair and regeneration processes. The review points out that various resident MAM proteins act as key regulators of Ca^2+^ exchange, ensuring proper intracellular Ca^2+^ shuttling and maintaining homeostasis. These processes are vital for several cellular functions, such as metabolic regulation, signal transduction, autophagy, and apoptosis. Importantly, it discusses how, under pathological conditions, the dysregulation of MAM-associated Ca^2+^ transport can lead to a series of harmful events, including mitochondrial dysfunction, Ca^2+^ overload in mitochondria, and increased production of ROS. We propose an innovative perspective that targeting MAM integrity and Ca^2+^ signaling could open up new avenues for therapy, positioning MAMs as a promising broad-spectrum target for treating neurodegenerative diseases. This comprehensive examination sheds light on the integral role of MAMs in cellular health and their potential as therapeutic targets, offering a fresh outlook on strategies for managing neurodegenerative disorders.

Building upon previous research, this review proposes that MAMs themselves are not the primary trigger of disease. Rather, they act as a pathogenic hub through which disease-associated factors exert their detrimental effects. Proteins intrinsically linked to familial neurodegeneration, such as α-syn and TDP-43, serve as potent disruptors of MAM dynamics through both direct and indirect mechanisms. While MAMs are unequivocally recognized as indispensable for neuronal function, their molecular complexity remains incompletely elucidated. Nevertheless, recent significant advances have begun to delineate key determinants governing MAM architecture, regulation, and functionality. Compelling evidence strongly supports the VAPB-PTPIP51 protein interaction as a genuine structural tether essential for MAM integrity. In contrast, the role of Mitofusin 2, initially proposed as an ER-mitochondrial tether, remains ambiguous; current understanding suggests it may primarily modulate MAM activity rather than constituting a core structural linker. Conversely, the Sig-1R functions as a positive regulator of MAM activity. By stabilizing and potentiating the activity of the IP3R, Sig-1R enhances ER-to-mitochondria Ca^2+^ transfer. Notably, pharmacological activation of Sig-1R ameliorates cellular deficits in models of ALS, underscoring the significant therapeutic potential of targeting ER-mitochondrial signaling pathways. A particularly striking observation is the emergence of disease-specific patterns in MAM-related Ca^2+^ flux dysregulation. Evidence indicates an upregulation of this flux in AD, contrasting with a downregulation observed in PD and ALS. This imbalance, whether excessive or insufficient Ca^2+^ transfer, acts as a potent pathogenic amplifier within each disease context, significantly exacerbating the core pathogenic cascades by further amplifying mitochondrial dysfunction. Consequently, strategies aimed at modulating MAM integrity and fine-tuning Ca^2+^ crosstalk represent a promising unified therapeutic approach to combat neurodegeneration. A growing body of research robustly demonstrates that dysregulation of ER-mitochondrial Ca^2+^ flux constitutes a near-universal hallmark across virtually all neurodegenerative diseases. However, the nature of this dysregulation exhibits specificity: it is typically increased in AD but decreased in PD and ALS, functioning effectively as a pathogenic amplifier within each distinct disorder. Furthermore, studies in AD reveal an added layer of complexity, where MAM-mediated Ca^2+^ transport often exhibits biphasic regulation during disease progression, suggesting potential compensatory mechanisms or stage-dependent roles. Contradictory findings regarding the involvement of MAM-associated proteins like Mitofusin 2 and Parkin in PD further highlight the inherent complexity of studying inter-organellar communication. These discrepancies underscore the critical need for more precise characterization of membrane contact site dynamics throughout the course of neurodegeneration. Importantly, these observations also reveal potential therapeutic targets. For instance, modulating the physical distance between the ER and mitochondria, potentially reducing it in specific contexts, emerges as a key mechanistic feature worth exploring in therapeutic strategies for both AD and PD. Current research efforts focused on the structural and functional characterization of MAMs remain significantly inadequate, presenting substantial scientific challenges. The inherent structural and functional complexity of MAMs, coupled with their fundamental role in cellular homeostasis, warrants intensified investigation. Simultaneously, Ca^2+^, acting as a pivotal second messenger in intra- and inter-cellular signaling, demands further rigorous study to elucidate its precise relationship with disease progression. A critical gap exists in understanding the compensatory mechanisms governing MAM-mediated Ca^2+^ flux during the pathological continuum. Deciphering these adaptive responses holds exceptional significance for identifying viable therapeutic targets in neurodegenerative diseases.

The role of MAMs in Ca^2+^ transport and signaling is increasingly recognized as a critical factor in aging and neurodegenerative diseases. The dynamic nature of MAMs, including their protein composition and interactions with the ER and mitochondria, underscores the need for advanced methodologies to study these structures in real-time and across different stages of disease progression. Current research highlights the importance of MAM-mediated Ca^2+^ signaling in processes vital for neuronal health, such as synaptic plasticity, neuroregeneration, and repair mechanisms following neural damage. However, the transient residency of certain proteins within MAMs presents challenges for traditional proteomic analyses, often leading to incomplete characterizations of the MAM proteome in diverse cell populations or tissues. Furthermore, a significant gap exists in our methodological toolkit—there is a lack of longitudinal observation techniques that can monitor ER-mitochondria interactions dynamically over clinically relevant timescales. This limitation hampers our understanding of how MAMs influence the pathophysiology of various neurodegenerative disorders and their progression through different stages. Despite the critical role that MAMs play, the development of therapeutics targeting these structures remains limited. Few compounds have progressed to clinical trials, with Blarcamesine (ANAVEX2-73), a Sigma-1 receptor agonist, being one of the rare candidates in phase III evaluation. There is a pressing need for innovative drug discovery efforts that leverage a variety of sources—natural products, small molecules, traditional medicine, and targeted biologics—to create agents that can effectively modulate MAM function.

The insights gained from investigating MAMs in the context of neurodegeneration are not confined to a single disease. The principles governing organelle interactions and neuronal fate have broader implications and may inform therapeutic strategies for a range of conditions affecting neural circuits, including traumatic brain injury and spinal cord injury. In summary, a comprehensive exploration of the role of MAMs in Ca^2+^ transport and its implications for neurodegeneration is crucial. This research not only has the potential to inform therapeutic strategies for Alzheimer’s disease and Parkinson’s disease but also offers a framework for addressing various neural circuit disruptions across multiple neurological conditions. Accelerating efforts in this domain is essential for the development of targeted and effective interventions aimed at enhancing neuronal health and resilience in the face of degenerative changes.

## Additional files:

***Additional Table 1:*** *Key targets of mitochondria-associated endoplasmic reticulum membranes in neurodegenerative disorders: mechanisms and druggability challenges.*

***Additional Table 2:*** *Active clinical trials targeting mitochondria-associated endoplasmic reticulum membranes-related pathways in neurodegenerative disorders (North America).*

***Additional Table 3:*** *FDA-related approval projects.*

***Additional Table 4:*** *NIH-funded projects targeting MAMs mechanisms.*

## Data Availability

*All relevant data are within the paper and its Additional files.*

## References

[R1] Agrawal RR, Montesinos J, Larrea D, Area-Gomez E, Pera M (2020). The silence of the fats: a MAM’s story about Alzheimer. Neurobiol Dis.

[R2] Aguilar-Hernández L, Alejandre R, César Morales-Medina J, Iannitti T, Flores G (2023). Cellular mechanisms in brain aging: Focus on physiological and pathological aging. J Chem Neuroanat.

[R3] Ahumada-Castro U, Puebla-Huerta A, Cuevas-Espinoza V, Lovy A, Cardenas JC (2021). Keeping zombies alive: The ER-mitochondria Ca(2+) transfer in cellular senescence. Biochim Biophys Acta Mol Cell Res.

[R4] Andronie-Cioara FL, Ardelean AI, Nistor-Cseppento CD, Jurcau A, Jurcau MC, Pascalau N, Marcu F (2023). Molecular mechanisms of neuroinflammation in aging and Alzheimer’s disease progression. Int J Mol Sci.

[R5] Area-Gomez E, de Groof A, Bonilla E, Montesinos J, Tanji K, Boldogh I, Pon L, Schon EA (2018). A key role for MAM in mediating mitochondrial dysfunction in Alzheimer disease. Cell Death Dis.

[R6] Area-Gomez E, Schon EA (2024). Towards a unitary hypothesis of Alzheimer’s disease pathogenesis. J Alzheimers Dis.

[R7] Argueti-Ostrovsky S, Barel S, Kahn J, Israelson A (2024). VDAC1: a key player in the mitochondrial landscape of neurodegeneration. Biomolecules.

[R8] Atakpa-Adaji P, Ivanova A (2023). IP (3)R at ER-mitochondrial contact sites: beyond the IP (3)R-GRP75-VDAC1 Ca (2+) funnel. Contact (Thousand Oaks).

[R9] Bai P, Gomm A, Yoo CH, Mondal P, Lobo FM, Meng H, Zhou Y, Xie W, Wey HY, Tanzi RE, Zhang C, Wang C, Lan Y (2025). Development of carbon-11 labeled pyrimidine derivatives as novel positron emission tomography (pet) agents enabling brain Sigma-1 receptor imaging. Adv Sci.

[R10] Banarase TA, Sammeta SS, Wankhede NL, Mangrulkar SV, Rahangdale SR, Aglawe MM, Taksande BG, Upaganlawar AB, Umekar MJ, Kale MB (2023). Mitophagy regulation in aging and neurodegenerative disease. Biophys Rev.

[R11] Basso V, Marchesan E, Ziviani E (2020). A trio has turned into a quartet: DJ–1 interacts with the IP3R-Grp75-VDAC complex to control ER-mitochondria interaction. Cell Calcium.

[R12] Bassot A (2023). The endoplasmic reticulum kinase PERK interacts with the oxidoreductase ero1 to metabolically adapt mitochondria. Cell Rep.

[R13] Belosludtsev KN, Sharipov RR, Boyarkin DP, Belosludtseva NV, Dubinin MV, Krasilnikova IA, Bakaeva ZV, Zgodova AE, Pinelis VG, Surin AM (2021). The effect of ds16570511, a new inhibitor of mitochondrial calcium uniporter, on calcium homeostasis, metabolism, and functional state of cultured cortical neurons and isolated brain mitochondria. Biochim Biophys Acta Gen Subj.

[R14] Ben-Hail D, Shoshan-Barmatz V (2016). VDAC1-interacting anion transport inhibitors inhibit VDAC1 oligomerization and apoptosis. Biochim Biophys Acta.

[R15] Berenguer-Escuder C, Grossmann D, Massart F, Antony P, Burbulla LF, Glaab E, Imhoff S, Trinh J, Seibler P, Grünewald A, Krüger R (2019). Variants in miro1 cause alterations of ER-mitochondria contact sites in fibroblasts from Parkinson’s disease patients. J Clin Med.

[R16] Bernardi P, Gerle C, Halestrap AP, Jonas EA, Karch J, Mnatsakanyan N, Pavlov E, Sheu SS, Soukas AA (2023). Identity, structure, and function of the mitochondrial permeability transition pore: controversies, consensus, recent advances, and future directions. Cell Death Differ.

[R17] Buonvicino D, Ranieri G, Guasti D, Pistolesi A, La Rocca AI, Rapizzi E, Chiarugi A (2023). Early derangement of axonal mitochondria occurs in a mouse model of progressive but not relapsing-remitting multiple sclerosis. Neurobiol Dis.

[R18] Burley S, Beccano-Kelly DA, Talbot K, Llana OC, Wade-Martins R (2022). Hyperexcitability in young iPSC-derived C9ORF72 mutant motor neurons is associated with increased intracellular calcium release. Sci Rep.

[R19] Calì T, Ottolini D, Vicario M, Catoni C, Vallese F, Cieri D, Barazzuol L, Brini M (2019). splitGFP technology reveals dose-dependent ER-mitochondria interface modulation by α-synuclein A53T and A30P mutants. Cells.

[R20] Cartes-Saavedra B, Ghosh A, Hajnóczky G (2025). The roles of mitochondria in global and local intracellular calcium signalling. Nat Rev Mol Cell Biol.

[R21] Cefis M, Dargegen M, Marcangeli V, Taherkhani S, Dulac M, Leduc-Gaudet JP, Mayaki D, Hussain SNA, Gouspillou G (2024). Mfn2 overexpression in skeletal muscles of young and old mice causes a mild hypertrophy without altering mitochondrial respiration and h (2)o (2) emission. Acta Physiol (Oxf).

[R22] Chen X, Mi L, Gu G, Gao X, Gao X, Shi M, Chai Y, Chen F, Yang W, Zhang J (2022). Dysfunctional endoplasmic reticulum-mitochondrion coupling is associated with endoplasmic reticulum stress-induced apoptosis and neurological deficits in a rodent model of severe head injury. J Neurotrauma.

[R23] Chen Y, Podojil JR, Kunjamma RB, Jones J, Weiner M, Lin W, Miller SD, Popko B (2019). Sephin1, which prolongs the integrated stress response, is a promising therapeutic for multiple sclerosis. Brain.

[R24] Choi GE, Park JY, Park MR, Yoon JH, Han HJ (2023). Glucocorticoid enhances presenilin1-dependent Aβ production at er’s mitochondrial-associated membrane by downregulating rer1 in neuronal cells. Redox Biol.

[R25] Choi SY, Lopez-Gonzalez R, Krishnan G, Phillips HL, Li AN, Seeley WW, Yao WD, Almeida S, Gao FB (2019). C9ORF72-ALS/FTD-associated poly (gr) binds ATP5A1 and compromises mitochondrial function in vivo. Nat Neurosci.

[R26] Cieri D, Vicario M, Giacomello M, Vallese F, Filadi R, Wagner T, Pozzan T, Pizzo P, Scorrano L, Brini M, Calì T (2018). Splics: a split green fluorescent protein-based contact site sensor for narrow and wide heterotypic organelle juxtaposition. Cell Death Differ.

[R27] Cortes-Figueiredo F, Asseyer S, Chien C, Zimmermann HG, Ruprecht K, Schmitz-Hübsch T, Bellmann-Strobl J, Paul F, Morais VA (2024). Cd4 (+) T cell mitochondrial genotype in multiple sclerosis: a cross-sectional and longitudinal analysis. Sci Rep.

[R28] Crespillo-Casado A, Claes Z, Choy MS, Peti W, Bollen M, Ron D (2018). A sephin1-insensitive tripartite holophosphatase dephosphorylates translation initiation factor 2α. J Biol Chem.

[R29] Dafinca R, Barbagallo P, Farrimond L, Candalija A, Scaber J, Ababneh NA, Sathyaprakash C, Vowles J, Cowley SA, Talbot K (2020). Impairment of mitochondrial calcium buffering links mutations in C9ORF72 and tardbp in IPS-derived motor neurons from patients with ALS/FTD. Stem Cell Reports.

[R30] Dafinca R, Scaber J, Ababneh N, Lalic T, Weir G, Christian H, Vowles J, Douglas AG, Fletcher-Jones A, Browne C, Nakanishi M, Turner MR, Wade-Martins R, Cowley SA, Talbot K (2016). C9orf72 hexanucleotide expansions are associated with altered endoplasmic reticulum calcium homeostasis and stress granule formation in induced pluripotent stem cell-derived neurons from patients with amyotrophic lateral sclerosis and frontotemporal dementia. Stem Cells.

[R31] Danese A, Patergnani S, Bonora M, Wieckowski MR, Previati M, Giorgi C, Pinton P (2017). Calcium regulates cell death in cancer: roles of the mitochondria and mitochondria-associated membranes (MAMs). Biochim Biophys Acta Bioenerg.

[R32] D’Angelo D, Rizzuto R (2023). The mitochondrial calcium uniporter (MCU): molecular identity and role in human diseases. Biomolecules.

[R33] das Neves SP, Sousa JC, Magalhães R, Gao F, Coppola G, Mériaux S, Boumezbeur F, Sousa N, Cerqueira JJ, Marques F (2023). Astrocytes undergo metabolic reprogramming in the multiple sclerosis animal model. Cells.

[R34] De Mario A, D’Angelo D, Zanotti G, Raffaello A, Mammucari C (2023). The mitochondrial calcium uniporter complex-a play in five acts. Cell Calcium.

[R35] Degechisa ST, Dabi YT, Gizaw ST (2022). The mitochondrial associated endoplasmic reticulum membranes: a platform for the pathogenesis of inflammation-mediated metabolic diseases. Immun Inflamm Dis.

[R36] Degirmenci Y, Angelopoulou E, Georgakopoulou VE, Bougea A (2023). Cognitive impairment in Parkinson’s disease: an updated overview focusing on emerging pharmaceutical treatment approaches. Medicina (Kaunas).

[R37] Dematteis G (2024). ER-mitochondria distance is a critical parameter for efficient mitochondrial Ca (2+) uptake and oxidative metabolism. Commun Biol.

[R38] Denley MCS, Straub MS, Marcionelli G, Güra MA, Penton D, Delvendahl I, Poms M, Vekeriotaite B, Cherkaoui S, Conte F, von Meyenn F, Froese DS, Baumgartner MR (2025). Mitochondrial dysfunction drives a neuronal exhaustion phenotype in methylmalonic aciduria. Commun Biol.

[R39] Dentoni G, Castro-Aldrete L, Naia L, Ankarcrona M (2022). The potential of small molecules to modulate the mitochondria-endoplasmic reticulum interplay in Alzheimer’s disease. Front Cell Dev Biol.

[R40] Dou X, Fu Q, Long Q, Liu S, Zou Y, Fu D, Xu Q, Jiang Z, Ren X, Zhang G, Wei X, Li Q, Campisi J, Zhao Y, Sun Y (2023). PDK4-dependent hypercatabolism and lactate production of senescent cells promotes cancer malignancy. Nat Metab.

[R41] Drewes N, Fang X, Gupta N, Nie D (2025). Pharmacological and pathological implications of Sigma-1 receptor in neurodegenerative diseases. Biomedicines.

[R42] Ekundayo BE, Obafemi TO, Adewale OB, Obafemi BA, Oyinloye BE, Ekundayo SK (2024). Oxidative stress, endoplasmic reticulum stress and apoptosis in the pathology of Alzheimer’s disease. Cell Biochem Biophys.

[R43] Espay AJ, Lees AJ (2024). Loss of monomeric alpha-synuclein (synucleinopenia) and the origin of Parkinson’s disease. Parkinsonism Relat Disord.

[R44] Ette EI, Fadiran EO, Missling C, Hammond E (2025). The new big is small: Leveraging knowledge from small trials for rare disease drug development: blarcamesine for rett syndrome. Br J Clin Pharmacol.

[R45] Faitg J, Lacefield C, Davey T, White K, Laws R, Kosmidis S, Reeve AK, Kandel ER, Vincent AE, Picard M (2021). 3D neuronal mitochondrial morphology in axons, dendrites, and somata of the aging mouse hippocampus. Cell Rep.

[R46] Fan H, Li Y, Huang J, Jiang J, Feng F, Huang N, Luo Y (2025). DJ-1 in Parkinson’s disease: its important role at endoplasmic reticulum-mitochondria contact sites. Behav Brain Res.

[R47] Fernandes T, Domingues MR, Moreira PI, Pereira CF (2023). A perspective on the link between mitochondria-associated membranes (MAMs) and lipid droplets metabolism in neurodegenerative diseases. Biology (Basel).

[R48] Filadi R, Greotti E, Turacchio G, Luini A, Pozzan T, Pizzo P (2015). Mitofusin 2 ablation increases endoplasmic reticulum-mitochondria coupling. Proc Natl Acad Sci U S A.

[R49] Filadi R, Greotti E, Turacchio G, Luini A, Pozzan T, Pizzo P (2016). Presenilin 2 modulates endoplasmic reticulum-mitochondria coupling by tuning the antagonistic effect of mitofusin 2. Cell Rep.

[R50] Gambardella J, Morelli MB, Wang X, Castellanos V, Mone P, Santulli G (2021). The discovery and development of IP3 receptor modulators: an update. Expert Opin Drug Discov.

[R51] Gao P, Yan Z, Zhu Z (2020). Mitochondria-associated endoplasmic reticulum membranes in cardiovascular diseases. Front Cell Dev Biol.

[R52] Garbincius JF, Elrod JW (2022). Mitochondrial calcium exchange in physiology and disease. Physiol Rev.

[R53] Gasmi A, Bjørklund G, Mujawdiya PK, Semenova Y, Piscopo S, Peana M (2024). Coenzyme q (10) in aging and disease. Crit Rev Food Sci Nutr.

[R54] Ge WD, Du TT, Wang CY, Sun LN, Wang YQ (2024). Calcium signaling crosstalk between the endoplasmic reticulum and mitochondria, a new drug development strategies of kidney diseases. Biochem Pharmacol.

[R55] Gomez-Suaga P, Paillusson S, Stoica R, Noble W, Hanger DP, Miller CCJ (2017). The ER-mitochondria tethering complex VAPB-PTPIP51 regulates autophagy. Curr Biol.

[R56] Groh J, Simons M (2025). White matter aging and its impact on brain function. Neuron.

[R57] Gülow K, Tümen D, Heumann P, Schmid S, Kandulski A, Müller M, Kunst C (2024). Unraveling the role of reactive oxygen species in T lymphocyte signaling. Int J Mol Sci.

[R58] Guo M, Liu R, Zhang F, Qu J, Yang Y, Li X (2024). A new perspective on liver diseases: Focusing on the mitochondria-associated endoplasmic reticulum membranes. Pharmacol Res.

[R59] Guo Y (2023). Small molecule agonist of mitochondrial fusion repairs mitochondrial dysfunction. Nat Chem Biol.

[R60] Hampel H, Williams C, Etcheto A, Goodsaid F, Parmentier F, Sallantin J, Kaufmann WE, Missling CU, Afshar M (2020). A precision medicine framework using artificial intelligence for the identification and confirmation of genomic biomarkers of response to an Alzheimer’s disease therapy: analysis of the blarcamesine (anavex2-73) phase 2a clinical study. Alzheimers Dement.

[R61] Han J, Park H, Maharana C, Gwon AR, Park J, Baek SH, Bae HG, Cho Y, Kim HK, Sul JH, Lee J, Kim E, Kim J, Cho Y, Park S, Palomera LF, Arumugam TV, Mattson MP, Jo DG (2021). Alzheimer’s disease-causing presenilin-1 mutations have deleterious effects on mitochondrial function. Theranostics.

[R62] Hasan P, Berezhnaya E, Rodríguez-Prados M, Weaver D, Bekeova C, Cartes-Saavedra B, Birch E, Beyer AM, Santos JH, Seifert EL, Elrod JW, Hajnóczky G (2024). MICU1 and MICU2 control mitochondrial calcium signaling in the mammalian heart. Proc Natl Acad Sci U S A.

[R63] He Q, Qu M, Shen T, Su J, Xu Y, Xu C, Barkat MQ, Cai J, Zhu H, Zeng LH, Wu X (2023). Control of mitochondria-associated endoplasmic reticulum membranes by protein s-palmitoylation: Novel therapeutic targets for neurodegenerative diseases. Ageing Res Rev.

[R64] Heneka MT (2025). Neuroinflammation in Alzheimer disease. Nat Rev Immunol.

[R65] Hibino M, Maeki M, Tokeshi M, Ishitsuka Y, Harashima H, Yamada Y (2023). A system that delivers an antioxidant to mitochondria for the treatment of drug-induced liver injury. Sci Rep.

[R66] Hoogerheide DP, Gurnev PA, Rostovtseva TK, Bezrukov SM (2022). Voltage-activated complexation of α-synuclein with three diverse β-barrel channels: VDAC, mspa, and α-hemolysin. Proteomics.

[R67] Houtman J, Freitag K, Gimber N, Schmoranzer J, Heppner FL, Jendrach M (2019). Beclin1-driven autophagy modulates the inflammatory response of microglia via NLRP3. EMBO J.

[R68] Hu Y, Chen H, Zhang L, Lin X, Li X, Zhuang H, Fan H, Meng T, He Z, Huang H, Gong Q, Zhu D, Xu Y, He P, Li L, Feng D (2021). The ampk-mfn2 axis regulates MAM dynamics and autophagy induced by energy stresses. Autophagy.

[R69] Huang CY, Deng JS, Huang WC, Jiang WP, Huang GJ (2020). Attenuation of lipopolysaccharide-induced acute lung injury by hispolon in mice, through regulating the TLR4/PI3K/Akt/mTOR and Keap1/Nrf2/HO-1 pathways, and suppressing oxidative stress-mediated ER stress-induced apoptosis and autophagy. Nutrients.

[R70] Igci M, Baysan M, Yigiter R, Ulasli M, Geyik S, Bayraktar R, Bozgeyik İ, Bozgeyik E, Bayram A, Cakmak EA (2016). Gene expression profiles of autophagy-related genes in multiple sclerosis. Gene.

[R71] Jadiya P, Kolmetzky DW, Tomar D, Di Meco A, Lombardi AA, Lambert JP, Luongo TS, Ludtmann MH, Praticò D, Elrod JW (2019). Impaired mitochondrial calcium efflux contributes to disease progression in models of Alzheimer’s disease. Nat Commun.

[R72] Jin H, Yang Y, Zhu X, Zhou Y, Xu Y, Li J, Qi C, Shao X, Wu J, Wu S, Cai H, Gu L, Mou S, Ni Z, Li S, Lin Q (2024). DDRGK1-mediated ER-phagy attenuates acute kidney injury through ER-stress and apoptosis. Cell Death Dis.

[R73] Johri A, Chandra A (2021). Connection lost, MAM: Errors in ER-mitochondria connections in neurodegenerative diseases. Brain Sci.

[R74] Kaye SD, Goyani S, Tomar D (2024). MICU1’s calcium sensing beyond mitochondrial calcium uptake. Biochim Biophys Acta Mol Cell Res.

[R75] Kirschner P, Pawlitzki M, Hartung HP, Meuth SG (2025). Immunology of multiple sclerosis: an update. Curr Opin Neurol.

[R76] Kmita H, Messina AA, De Pinto V (2023). VDAC as a cellular hub: docking molecules and interactions. Int J Mol Sci.

[R77] Kou JJ, Shi JZ, He YY, Hao JJ, Zhang HY, Luo DM, Song JK, Yan Y, Xie XM, Du GH, Pang XB (2022). Luteolin alleviates cognitive impairment in Alzheimer’s disease mouse model via inhibiting endoplasmic reticulum stress-dependent neuroinflammation. Acta Pharmacol Sin.

[R78] Larrañaga-SanMiguel A, Bengoa-Vergniory N, Flores-Romero H (2025). Crosstalk between mitochondria-ER contact sites and the apoptotic machinery as a novel health meter. Trends Cell Biol.

[R79] Larrea D, Tamucci KA, Kabra K, Velasco KR, Yun TD, Pera M, Montesinos J, Agrawal RR, Paradas C, Smerdon JW, Lowry ER, Stepanova A, Yoval-Sanchez B, Galkin A, Wichterle H, Area-Gomez E (2025). Altered mitochondria-associated ER membrane (MAM) function shifts mitochondrial metabolism in amyotrophic lateral sclerosis (ALS). Nat Commun.

[R80] Leal NS, Dentoni G, Schreiner B, Naia L, Piras A, Graff C, Cattaneo A, Meli G, Hamasaki M, Nilsson P, Ankarcrona M (2020). Amyloid β-peptide increases mitochondria-endoplasmic reticulum contact altering mitochondrial function and autophagosome formation in Alzheimer’s disease-related models. Cells.

[R81] Lebeau J, Saunders JM, Moraes VWR, Madhavan A, Madrazo N, Anthony MC, Wiseman RL (2018). The PERK arm of the unfolded protein response regulates mitochondrial morphology during acute endoplasmic reticulum stress. Cell Rep.

[R82] Lemos FO, de Ridder I, Wagner L, Bootman MD, Bultynck G, Yule DI, Parys JB (2024). Tetrameric, active PKM2 inhibits IP (3) receptors, potentially requiring GRP75 as an additional interaction partner. Biochim Biophys Acta Mol Cell Res.

[R83] Li C, Li L, Yang M, Yang J, Zhao C, Han Y, Zhao H, Jiang N, Wei L, Xiao Y, Liu Y, Xiong X, Xi Y, Luo S, Deng F, Chen W, Yuan S, Zhu X, Xiao L, Sun L (2022). Pacs-2 ameliorates tubular injury by facilitating endoplasmic reticulum-mitochondria contact and mitophagy in diabetic nephropathy. Diabetes.

[R84] Li C, Sun J, Zhang X, Zhou M, Gan X (2023). Implications of MCU complex in metabolic diseases. FASEB J.

[R85] Li S, Jin SX (2025). Beta 2-adrenergic pathway combats Alzheimer’s disease: Restoring cognition and synaptic integrity. Neural Regen Res.

[R86] Li X, Gou F, Zhu J, Lin Q, Yu M, Tu X, Hong Q, Hu C (2024). Deoxynivalenol induced intestinal barrier injury, mitochondrial dysfunction and calcium overload by inositol 1, 4, 5-triphosphate receptors (ip3rs)-mitochondrial calcium uniporter (MCU) calcium axis. Sci Total Environ.

[R87] Li X, Zhao X, Qin Z, Li J, Sun B, Liu L (2025). Regulation of calcium homeostasis in endoplasmic reticulum-mitochondria crosstalk: Implications for skeletal muscle atrophy. Cell Commun Signal.

[R88] Li Y, Tian X, Luo J, Bao T, Wang S, Wu X (2024). Molecular mechanisms of aging and anti-aging strategies. Cell Commun Signal.

[R89] Li Z, Huang S, Li H, Liu Q, Lu J, Liu P, Wu Z (2025). Perk’s novel agonist protects against myocardial ischemia-reperfusion injury by modulating ER-mitochondria contacts and phosphatidic acid transport. Int J Cardiol.

[R90] Li Z, Ran Q, Qu C, Hu S, Cui S, Zhou Y, Shen B, Yang B (2025). Sigma-1 receptor activation attenuates dox-induced cardiotoxicity by alleviating endoplasmic reticulum stress and mitochondrial calcium overload via PERK and IP3R-VDAC1-MCU signaling pathways. Biol Direct.

[R91] Liang P, Le W (2015). Role of autophagy in the pathogenesis of multiple sclerosis. Neurosci Bull.

[R92] Liu Y, Jin M, Wang Y, Zhu J, Tan R, Zhao J, Ji X, Jin C, Jia Y, Ren T, Xing J (2020). MCU-induced mitochondrial calcium uptake promotes mitochondrial biogenesis and colorectal cancer growth. Signal Transduct Target Ther.

[R93] López-Muguruza E, Matute C (2023). Alterations of oligodendrocyte and myelin energy metabolism in Multiple Sclerosis. Int J Mol Sci.

[R94] Lou G, Palikaras K, Lautrup S, Scheibye-Knudsen M, Tavernarakis N, Fang EF (2020). Mitophagy and neuroprotection. Trends Mol Med.

[R95] Luo R, Gourriérec PL, Antigny F, Bedouet K, Domenichini S, Gomez AM, Benitah JP, Sabourin J (2024). STIM2 variants regulate Orai1/TRPC1/TRPC4-mediated store-operated Ca (2+) entry and mitochondrial Ca (2+) homeostasis in cardiomyocytes. Cell Calcium.

[R96] Lv Y, Chen L, Li L, Liao Z, Fang Z, Cheng L, Peng F (2025). The structure and function of mitofusin 2 and its role in cardiovascular disease through mediating mitochondria-associated endoplasmic reticulum membranes. Front Cardiovasc Med.

[R97] Macfarlane S (2025). Blarcamesine for the treatment of early Alzheimer’s disease: results from the ANAVEX2-73-AD-004 phase iib/iii trial. J Prev Alzheimers Dis.

[R98] Mahakud AK, Shaikh J, Rifa Iqbal VV, Gupta A, Tiwari A, Saleem M (2022). Amyloids on membrane interfaces: implications for neurodegeneration. J Membr Biol.

[R99] Mao H, Chen W, Chen L, Li L (2022). Potential role of mitochondria-associated endoplasmic reticulum membrane proteins in diseases. Biochem Pharmacol.

[R100] Mao Y, Wang Z, Yao C, Zeng Q, Cheng W, Zhang S, Chen S, Sheng C (2022). The food and drug administration-approved antipsychotic drug trifluoperazine, a calmodulin antagonist, inhibits viral replication through PERK-eif2α axis. Front Microbiol.

[R101] Marinho D, Ferreira IL, Lorenzoni R, Cardoso SM, Santana I, Rego AC (2023). Reduction of class I histone deacetylases ameliorates ER-mitochondria cross-talk in Alzheimer’s disease. Aging Cell.

[R102] Markovinovic A, Martín-Guerrero SM, Mórotz GM, Salam S, Gomez-Suaga P, Paillusson S, Greig J, Lee Y, Mitchell JC, Noble W, Miller CCJ (2024). Stimulating VAPB-PTPIP51 ER-mitochondria tethering corrects FTD/ALS mutant TDP43 linked Ca (2+) and synaptic defects. Acta Neuropathol Commun.

[R103] Maurice T (2025). Prevention of memory impairment and hippocampal injury with blarcamesine in an Alzheimer’s disease model. Neurosci Lett.

[R104] McLelland GL, Goiran T, Yi W, Dorval G, Chen CX, Lauinger ND, Krahn AI, Valimehr S, Rakovic A, Rouiller I, Durcan TM, Trempe JF, Fon EA (2018). Mfn2 ubiquitination by PINK1/parkin gates the p97-dependent release of ER from mitochondria to drive mitophagy. Elife.

[R105] Melo Dos Santos LS, Trombetta-Lima M, Eggen B, Demaria M (2024). Cellular senescence in brain aging and neurodegeneration. Ageing Res Rev.

[R106] Mercer RCC, Le NTT, Fraser DG, Houser MCQ, Beeler AB, Harris DA (2024). Sigma receptor ligands are potent antiprion compounds that act independently of sigma receptor binding. ACS Chem Neurosci.

[R107] Mori F, Nakamura Y, Miki Y, Tanji K, Kon T, Tomiyama M, Kakita A, Wakabayashi K (2022). Alteration of vesicle-associated membrane protein-binding protein b in α-synuclein aggregates in lewy body disease. J Neuropathol Exp Neurol.

[R108] Morris JL, Gillet G, Prudent J, Popgeorgiev N (2021). Bcl-2 family of proteins in the control of mitochondrial calcium signalling: an old chap with new roles. Int J Mol Sci.

[R109] Müller M, Ahumada-Castro U, Sanhueza M, Gonzalez-Billault C, Court FA, Cárdenas C (2018). Mitochondria and calcium regulation as basis of neurodegeneration associated with aging. Front Neurosci.

[R110] Munguia-Galaviz FJ, Miranda-Diaz AG, Cardenas-Sosa MA, Echavarria R (2023). Sigma-1 receptor signaling: in search of new therapeutic alternatives for cardiovascular and renal diseases. Int J Mol Sci.

[R111] Naia L (2021). Neuronal cell-based high-throughput screen for enhancers of mitochondrial function reveals luteolin as a modulator of mitochondria-endoplasmic reticulum coupling. BMC Biol.

[R112] Naón D, Hernández-Alvarez MI, Shinjo S, Wieczor M, Ivanova S, Martins de Brito O, Quintana A, Hidalgo J, Palacín M, Aparicio P, Castellanos J, Lores L, Sebastián D, Fernández-Veledo S, Vendrell J, Joven J, Orozco M, Zorzano A, Scorrano L (2023). Splice variants of mitofusin 2 shape the endoplasmic reticulum and tether it to mitochondria. Science.

[R113] Narendra DP, Youle RJ (2024). The role of pink1-parkin in mitochondrial quality control. Nat Cell Biol.

[R114] Newton K, Strasser A, Kayagaki N, Dixit VM (2024). Cell death. Cell.

[R115] Nhu NT, Xiao SY, Liu Y, Kumar VB, Cui ZY, Lee SD (2021). Neuroprotective effects of a small mitochondrially-targeted tetrapeptide elamipretide in neurodegeneration. Front Integr Neurosci.

[R116] Obara CJ, Nixon-Abell J, Moore AS, Riccio F, Hoffman DP, Shtengel G, Xu CS, Schaefer K, Pasolli HA, Masson JB, Hess HF, Calderon CP, Blackstone C, Lippincott-Schwartz J (2024). Motion of vapb molecules reveals ER-mitochondria contact site subdomains. Nature.

[R117] Parrado-Fernández C, Schneider B, Ankarcrona M, Conti MM, Cookson MR, Kivipelto M, Cedazo-Mínguez Á, Sandebring-Matton A (2018). Reduction of pink1 or DJ-1 impair mitochondrial motility in neurites and alter ER-mitochondria contacts. J Cell Mol Med.

[R118] Patergnani S, Bonora M, Ingusci S, Previati M, Marchi S, Zucchini S, Perrone M, Wieckowski MR, Castellazzi M, Pugliatti M, Giorgi C, Simonato M, Pinton P (2021). Antipsychotic drugs counteract autophagy and mitophagy in multiple sclerosis. Proc Natl Acad Sci U S A.

[R119] Pedriali G, Rimessi A, Sbano L, Giorgi C, Wieckowski MR, Previati M, Pinton P (2017). Regulation of endoplasmic reticulum-mitochondria Ca (2+) transfer and its importance for anti-cancer therapies. Front Oncol.

[R120] Pelizzari Raymundo D, Eriksson LA, Chevet E, Guillory X (2022). Structure-based drug discovery of IRE1 modulators. Methods Mol Biol.

[R121] Perea V, Cole C, Lebeau J, Dolina V, Baron KR, Madhavan A, Kelly JW, Grotjahn DA, Wiseman RL (2023). PERK signaling promotes mitochondrial elongation by remodeling membrane phosphatidic acid. EMBO J.

[R122] Peviani M, Salvaneschi E, Bontempi L, Petese A, Manzo A, Rossi D, Salmona M, Collina S, Bigini P, Curti D (2014). Neuroprotective effects of the Sigma-1 receptor (s1r) agonist PRE-084, in a mouse model of motor neuron disease not linked to SOD1 mutation. Neurobiol Dis.

[R123] Pittalà MGG, Reina S, Nibali SC, Cucina A, Cubisino SAM, Cunsolo V, Amodeo GF, Foti S, De Pinto V, Saletti R, Messina A (2022). Specific post-translational modifications of VDAC3 in ALS-SOD1 model cells identified by high-resolution mass spectrometry. Int J Mol Sci.

[R124] Ponneri Babuharisankar A, Kuo CL, Chou HY, Tangeda V, Fan CC, Chen CH, Kao YH, Lee AY (2023). Mitochondrial lon-induced mitophagy benefits hypoxic resistance via Ca (2+)-dependent Fundc1 phosphorylation at the ER-mitochondria interface. Cell Death Dis.

[R125] Popov LD (2023). Mitochondria as intracellular signalling organelles. An update. Cell Signal.

[R126] Puebla-Huerta A (2025). Calcium (Ca (2+)) fluxes at mitochondria-ER contact sites (MERCS) are a new target of senolysis in therapy-induced senescence (TIS). NPJ Aging.

[R127] Pukoli D, Vécsei L (2023). Smouldering lesion in ms: microglia, lymphocytes and pathobiochemical mechanisms. Int J Mol Sci.

[R128] Quessada C, Bouscary A, René F, Valle C, Ferri A, Ngo ST, Loeffler JP (2021). Skeletal muscle metabolism: origin or prognostic factor for Amyotrophic Lateral Sclerosis (ALS) development?. Cells.

[R129] Quinn PMJ, Moreira PI, Ambrósio AF, Alves CH (2020). Pink1/parkin signalling in neurodegeneration and neuroinflammation. Acta Neuropathol Commun.

[R130] Ramezani M, Wagenknecht-Wiesner A, Wang T, Holowka DA, Eliezer D, Baird BA (2023). Alpha synuclein modulates mitochondrial Ca (2+) uptake from ER during cell stimulation and under stress conditions. NPJ Parkinsons Dis.

[R131] Repici M, Giorgini F (2019). DJ-1 in Parkinson’s disease: clinical insights and therapeutic perspectives. J Clin Med.

[R132] Resende R, Fernandes T, Pereira AC, Marques AP, Pereira CF (2022). Endoplasmic reticulum-mitochondria contacts modulate reactive oxygen species-mediated signaling and oxidative stress in brain disorders: the key role of Sigma-1 receptor. Antioxid Redox Signal.

[R133] Righes G, Semenzato L, Koutsikos K, Zanato V, Pinton P, Giorgi C, Patergnani S (2025). The role of autophagy in the pathogenesis and treatment of multiple sclerosis. Autophagy Rep.

[R134] Rizzuto R, Pinton P, Carrington W, Fay FS, Fogarty KE, Lifshitz LM, Tuft RA, Pozzan T (1998). Close contacts with the endoplasmic reticulum as determinants of mitochondrial Ca2+ responses. Science.

[R135] Rodríguez-Prados M, Berezhnaya E, Castromonte MT, Menezes-Filho SL, Paillard M, Hajnóczky G (2023). MICU1 occludes the mitochondrial calcium uniporter in divalent-free conditions. Proc Natl Acad Sci U S A.

[R136] Rossini M, García-Casas P, Filadi R, Pizzo P (2021). Loosening ER-mitochondria coupling by the expression of the presenilin 2 loop domain. Cells.

[R137] Rummens J, Da Cruz S (2025). RNA-binding proteins in ALS and FTD: from pathogenic mechanisms to therapeutic insights. Mol Neurodegener.

[R138] Ryskamp D, Wu L, Wu J, Kim D, Rammes G, Geva M, Hayden M, Bezprozvanny I (2019). Pridopidine stabilizes mushroom spines in mouse models of Alzheimer’s disease by acting on the sigma-1 receptor. Neurobiol Dis.

[R139] Sakai S, Watanabe S, Komine O, Sobue A, Yamanaka K (2021). Novel reporters of mitochondria-associated membranes (MAM), mamtrackers, demonstrate MAM disruption as a common pathological feature in amyotrophic lateral sclerosis. FASEB J.

[R140] Sathyamurthy VH, Nagarajan Y, Parvathi VD (2024). Mitochondria-endoplasmic reticulum contact sites (MERCS): a new axis in neuronal degeneration and regeneration. Mol Neurobiol.

[R141] Schreiner B, Hedskog L, Wiehager B, Ankarcrona M (2015). Amyloid-β peptides are generated in mitochondria-associated endoplasmic reticulum membranes. J Alzheimers Dis.

[R142] Seegren PV, Harper LR, Downs TK, Zhao XY, Viswanathan SB, Stremska ME, Olson RJ, Kennedy J, Ewald SE, Kumar P, Desai BN (2023). Reduced mitochondrial calcium uptake in macrophages is a major driver of inflammaging. Nat Aging.

[R143] Sharma N, Patel C, Shenkman M, Kessel A, Ben-Tal N, Lederkremer GZ (2021). The Sigma-1 receptor is an ER-localized type II membrane protein. J Biol Chem.

[R144] Shi L, Wang M, Yu R, An Y, Wang X, Zhang Y, Shi Y, Han C, Liu J (2025). Sigma-1 receptor agonist PRE-084 increases bdnf by activating the erk/creb pathway to rescue learning and memory impairment caused by type II diabetes. Behav Brain Res.

[R145] Shi M, Chen F, Chen Z, Yang W, Yue S, Zhang J, Chen X (2021). Sigma-1 receptor: a potential therapeutic target for traumatic brain injury. Front Cell Neurosci.

[R146] Singh R, Kaur N, Choubey V, Dhingra N, Kaur T (2024). Endoplasmic reticulum stress and its role in various neurodegenerative diseases. Brain Res.

[R147] Siwecka N, Rozpędek-Kamińska W, Wawrzynkiewicz A, Pytel D, Diehl JA, Majsterek I (2021). The structure, activation and signaling of IRE1 and its role in determining cell fate. Biomedicines.

[R148] Skou LD, Johansen SK, Okarmus J, Meyer M (2024). Pathogenesis of DJ-1/park7-mediated Parkinson’s disease. Cells.

[R149] Smedley GD, Walker KE, Yuan SH (2021). The role of PERK in understanding development of neurodegenerative diseases. Int J Mol Sci.

[R150] Smilansky A, Dangoor L, Nakdimon I, Ben-Hail D, Mizrachi D, Shoshan-Barmatz V (2015). The voltage-dependent anion channel 1 mediates amyloid β toxicity and represents a potential target for Alzheimer disease therapy. J Biol Chem.

[R151] Smith HA, Thillaiappan NB, Rossi AM (2023). Ip (3) receptors: an “elementary” journey from structure to signals. Cell Calcium.

[R152] Strope TA, Wilkins HM (2024). The reciprocal relationship between amyloid precursor protein and mitochondrial function. J Neurochem.

[R153] Sun J, Jin S, Wang Z (2025). Mitoq alleviates mitochondria damage in sepsis-acute lung injury in a citrate synthase dependent manner. Inflamm Res.

[R154] Sushma, Mishra S, Kanchan S, Divakar A, Jha G, Sharma D, Kapoor R, Kumar Rath S (2023). Alcohol induces ER stress and apoptosis by inducing oxidative stress and disruption of calcium homeostasis in glial cells. Food Chem Toxicol.

[R155] Takebayashi S, Tanaka H, Hino S, Nakatsu Y, Igata T, Sakamoto A, Narita M, Nakao M (2015). Retinoblastoma protein promotes oxidative phosphorylation through upregulation of glycolytic genes in oncogene-induced senescent cells. Aging Cell.

[R156] Takeda K, Nagashima S, Shiiba I, Uda A, Tokuyama T, Ito N, Fukuda T, Matsushita N, Ishido S, Iwawaki T, Uehara T, Inatome R, Yanagi S (2019). Mitol prevents ER stress-induced apoptosis by ire1α ubiquitylation at ER-mitochondria contact sites. EMBO J.

[R157] Tambini MD, Pera M, Kanter E, Yang H, Guardia-Laguarta C, Holtzman D, Sulzer D, Area-Gomez E, Schon EA (2016). Apoe4 upregulates the activity of mitochondria-associated ER membranes. EMBO Rep.

[R158] Tanji C, Hashimoto M, Furuya T, Saito J, Miyamoto T, Koda M (2021). Sigma 1 receptor agonist cutamesine promotes plasticity of serotonergic boutons in lumbar enlargement in spinal cord injured rats. Neurosci Lett.

[R159] Tinu N, Sharma B, Rodarte D, Lakshmanaswamy R, Kumar S (2025). Interplay between brain-specific microRNAs and Alzheimer’s disease. Neural Regen Res.

[R160] Toyofuku T, Okamoto Y, Ishikawa T, Sasawatari S, Kumanogoh A (2020). Lrrk2 regulates endoplasmic reticulum-mitochondrial tethering through the PERK-mediated ubiquitination pathway. EMBO J.

[R161] Tur J, Pereira-Lopes S, Vico T, Marín EA, Muñoz JP, Hernández-Alvarez M, Cardona PJ, Zorzano A, Lloberas J, Celada A (2020). Mitofusin 2 in macrophages links mitochondrial ros production, cytokine release, phagocytosis, autophagy, and bactericidal activity. Cell Rep.

[R162] Vallese F, Barazzuol L, Maso L, Brini M, Calì T (2020). ER-mitochondria calcium transfer, organelle contacts and neurodegenerative diseases. Adv Exp Med Biol.

[R163] Vanderhaeghe S, Prerad J, Tharkeshwar AK, Goethals E, Vints K, Beckers J, Scheveneels W, Debroux E, Princen K, Van Damme P, Fivaz M, Griffioen G, Van Den Bosch L (2024). A pathogenic mutation in the ALS/FTD gene vcp induces mitochondrial hypermetabolism by modulating the permeability transition pore. Acta Neuropathol Commun.

[R164] Vazquez-Sanchez S, Tilkin B, Gasset-Rosa F, Zhang S, Piol D, McAlonis-Downes M, Artates J, Govea-Perez N, Verresen Y, Guo L, Cleveland DW, Shorter J, Da Cruz S (2024). Frontotemporal dementia-like disease progression elicited by seeded aggregation and spread of FUS. Mol Neurodegener.

[R165] Verfaillie T, Rubio N, Garg AD, Bultynck G, Rizzuto R, Decuypere JP, Piette J, Linehan C, Gupta S, Samali A, Agostinis P (2012). PERK is required at the ER-mitochondrial contact sites to convey apoptosis after ros-based ER stress. Cell Death Differ.

[R166] Villard V, Espallergues J, Keller E, Vamvakides A, Maurice T (2011). Anti-amnesic and neuroprotective potentials of the mixed muscarinic receptor/sigma 1 (σ1) ligand anavex2-73, a novel aminotetrahydrofuran derivative. J Psychopharmacol.

[R167] Wang B, Han J, Elisseeff JH, Demaria M (2024). The senescence-associated secretory phenotype and its physiological and pathological implications. Nat Rev Mol Cell Biol.

[R168] Wang N, Wang C, Zhao H, He Y, Lan B, Sun L, Gao Y (2021). The MAMs structure and its role in cell death. Cells.

[R169] Wang N, Wang X, Lan B, Gao Y, Cai Y (2025). DRP1, fission and apoptosis. Cell Death Discov.

[R170] Wang Q, Duan L, Li X, Wang Y, Guo W, Guan F, Ma S (2022). Glucose metabolism, neural cell senescence and Alzheimer’s disease. Int J Mol Sci.

[R171] Wang T, Jia H (2023). The Sigma receptors in Alzheimer’s disease: New potential targets for diagnosis and therapy. Int J Mol Sci.

[R172] Wang X, Jia J (2023). Magnolol improves Alzheimer’s disease-like pathologies and cognitive decline by promoting autophagy through activation of the AMPK/mTOR/ULK1 pathway. Biomed Pharmacother.

[R173] Wang Y, Wang Y, Yin H, Xiao Z, Ren Z, Ma X, Zhang J, Fu X, Zhang F, Zeng L (2025). Bi1 activates autophagy and mediates TDP43 to regulate ALS pathogenesis. Mol Neurobiol.

[R174] Wang ZJ, Zhao F, Wang CF, Zhang XM, Xiao Y, Zhou F, Wu MN, Zhang J, Qi JS, Yang W (2019). Xestospongin c, a reversible IP3 receptor antagonist, alleviates the cognitive and pathological impairments in APP/PS1 mice of Alzheimer’s disease. J Alzheimers Dis.

[R175] Waseem M, Wang BD (2023). Promising strategy of mptp modulation in cancer therapy: an emerging progress and future insight. Int J Mol Sci.

[R176] Watanabe S, Ilieva H, Tamada H, Nomura H, Komine O, Endo F, Jin S, Mancias P, Kiyama H, Yamanaka K (2016). Mitochondria-associated membrane collapse is a common pathomechanism in sigmar1- and SOD1-linked ALS. EMBO Mol Med.

[R177] Watanabe S, Horiuchi M, Murata Y, Komine O, Kawade N, Sobue A, Yamanaka K (2023). Sigma-1 receptor maintains ATAD3A as a monomer to inhibit mitochondrial fragmentation at the mitochondria-associated membrane in amyotrophic lateral sclerosis. Neurobiol Dis.

[R178] Watanabe S, Murata Y, Oka Y, Oiwa K, Horiuchi M, Iguchi Y, Komine O, Sobue A, Katsuno M, Ogi T, Yamanaka K (2023). Mitochondria-associated membrane collapse impairs tbk1-mediated proteostatic stress response in ALS. Proc Natl Acad Sci U S A.

[R179] Wu J, Xu W, Su Y, Wang GH, Ma JJ (2025). Targeting chaperone-mediated autophagy in neurodegenerative diseases: Mechanisms and therapeutic potential. Acta Pharmacol Sin.

[R180] Xu Y, Chen J, Chen J, Teng J (2022). Ei24 promotes cell adaption to ER stress by coordinating IRE1 signaling and calcium homeostasis. EMBO Rep.

[R181] Yang J, Shi X, Wang Y, Ma M, Liu H, Wang J, Xu Z (2023). Multi-target neuroprotection of thiazolidinediones on Alzheimer’s disease via neuroinflammation and ferroptosis. J Alzheimers Dis.

[R182] Yang Y, Jia X, Yang X, Wang J, Fang Y, Ying X, Zhang M, Wei J, Pan Y (2024). Targeting VDAC: a potential therapeutic approach for mitochondrial dysfunction in Alzheimer’s disease. Brain Res.

[R183] Yang Y, Chen M, Ding L, Liu J, Luo J, Yan R, Ning J, Xie S, Li X, Ren Z, Zhou R, Chen Z (2025). Mitochondria-associated endoplasmic reticulum membranes and calcium ion exchange: A novel direction for aging and neurodegenerative diseases. Neural Regen Res.

[R184] Yoo J (2022). Structural basis of Ca (2+) uptake by mitochondrial calcium uniporter in mitochondria: a brief review. BMB Rep.

[R185] Yu S, Zhang L, Liu C, Yang J, Zhang J, Huang L (2019). PACS2 is required for ox-LDL-induced endothelial cell apoptosis by regulating mitochondria-associated ER membrane formation and mitochondrial Ca (2+) elevation. Exp Cell Res.

[R186] Yu W, Li Y, Li Y, Hu J, Wu J, Chen X, Huang Y, Shi X (2025). Connexin43 contributes to Alzheimer’s disease by promoting the mitochondria-associated membrane-related autophagy inhibition. Mol Neurobiol.

[R187] Zampese E, Fasolato C, Kipanyula MJ, Bortolozzi M, Pozzan T, Pizzo P (2011). Presenilin 2 modulates endoplasmic reticulum (ER)-mitochondria interactions and Ca2+ cross-talk. Proc Natl Acad Sci U S A.

[R188] Zellmer JC, Tarantino MB, Kim M, Lomoio S, Maesako M, Hajnóczky G, Bhattacharyya R (2025). Stabilization of mitochondria-associated endoplasmic reticulum membranes regulates Aβ generation in a three-dimensional neural model of Alzheimer’s disease. Alzheimers Dement.

[R189] Zhang J, Tao J, Zhou Z, Pei W, Xiao Y, Guo Y, Gao J, Jiang C, Dai L, Zhang G, Tan C (2025). Current research on mitochondria-associated membranes in cardiovascular diseases (review). Mol Med Rep.

[R190] Zhang Y, Rao X, Wang J, Liu H, Wang Q, Wang X, Hua F, Guan X, Lin Y (2025). Mitochondria-associated membranes: a key point of neurodegenerative diseases. CNS Neurosci Ther.

[R191] Zhang YM, Wang YT, Wei RM, Li XY, Luo BL, Zhang JY, Zhang KX, Fang SK, Liu XC, Chen GH (2024). Mitochondrial antioxidant elamipretide improves learning and memory impairment induced by chronic sleep deprivation in mice. Brain Behav.

[R192] Zhao H, Chen S, Cao N, Wu W, Liu G, Gao J, Chen J, Li T, Lu D, Zeng L, Zhu H, Zhang W, Xia Q, Li T, Zhou T, Zhang XM, Li AL, Pan X (2025). Berberine is a novel mitochondrial calcium uniporter inhibitor that disrupts MCU-emre assembly. Adv Sci.

[R193] Zhao WB, Sheng R (2025). The correlation between mitochondria-associated endoplasmic reticulum membranes (MAMs) and Ca (2+) transport in the pathogenesis of diseases. Acta Pharmacol Sin.

[R194] Zhao Y, Chang J, Zhang B, Tong P, Wang C, Ran D, Su Y (2019). TLR-5 agonist salmonella abortus equi flagellin FliC enhances FliC-gd-based DNA vaccination against equine herpesvirus 1 infection. Arch Virol.

[R195] Zhao Y, Sun X, Hu D, Prosdocimo DA, Hoppel C, Jain MK, Ramachandran R, Qi X (2019). ATAD3A oligomerization causes neurodegeneration by coupling mitochondrial fragmentation and bioenergetics defects. Nat Commun.

[R196] Zhao Y, Hu D, Wang R, Sun X, Ropelewski P, Hubler Z, Lundberg K, Wang Q, Adams DJ, Xu R, Qi X (2022). ATAD3A oligomerization promotes neuropathology and cognitive deficits in Alzheimer’s disease models. Nat Commun.

[R197] Zhao Y, Chang YH, Ren HR, Lou M, Jiang FW, Wang JX, Chen MS, Liu S, Shi YS, Zhu HM, Li JL (2024). Phthalates induce neurotoxicity by disrupting the Mfn2-PERK axis-mediated endoplasmic reticulum-mitochondria interaction. J Agric Food Chem.

[R198] Zheng G, Wang L, Li X, Niu X, Xu G, Lv P (2021). Rapamycin alleviates cognitive impairment in murine vascular dementia: the enhancement of mitophagy by PI3K/akt/mTOR axis. Tissue Cell.

[R199] Zhou W, Graner M, Paucek P, Beseler C, Boisen M, Bubak A, Asturias F, George W, Graner A, Ormond D, Vollmer T, Alvarez E, Yu X (2023). Multiple sclerosis plasma igg aggregates induce complement-dependent neuronal apoptosis. Cell Death Dis.

[R200] Zhu R, Liu L, Mao T, Wang X, Li Y, Li T, Lv S, Zeng S, Fu N, Li N, Wang Y, Sun M, Zhang J (2024). Mfn2 regulates mitochondria and mitochondria-associated endoplasmic reticulum membrane function in neurodegeneration induced by repeated sevoflurane exposure. Exp Neurol.

[R201] Zhu S, Li X, Dang B, Wu F, Wang C, Lin C (2022). Lycium barbarum polysaccharide protects hacat cells from PM2. 5-induced apoptosis via inhibiting oxidative stress, ER stress and autophagy. Redox Rep.

[R202] Zhu X, Qin Z, Zhou M, Li C, Jing J, Ye W, Gan X (2024). The role of mitochondrial permeability transition in bone metabolism, bone healing, and bone diseases. Biomolecules.

